# Solvent-Based Extraction Recovers Phytochemicals from Medicinal Plants Demonstrating Anticancer and Chemopreventive Potential: A Review

**DOI:** 10.3390/molecules31071202

**Published:** 2026-04-04

**Authors:** Cecile Ojong, Samuel A. Besong, Alberta N. A. Aryee

**Affiliations:** Food Science & Biotechnology Program, Department of Human Ecology, College Agriculture, Science and Technology, Delaware State University, 1200 N DuPont Highway, Dover, DE 19901, USA

**Keywords:** medicinal plants, phytochemicals, solvent-based extraction techniques, bioactivity, anticancer agents, alternative therapies

## Abstract

Cancer remains a leading cause of morbidity and mortality globally, with current therapies often limited by toxicity, drug resistance, and reduced efficacy in advanced stages. Medicinal plants represent important sources of bioactive compounds (BACs) with anticancer and chemopreventive potential; however, their successful application is strongly influenced by extraction strategies that determine phytochemical recovery and downstream biological activity. This review evaluates solvent-based extraction techniques used to extract BACs from medicinal plants with reported anticancer properties, synthesizing peer-reviewed articles from PubMed and Google Scholar published between 2020 and 2025. Solvent-based methods, including Soxhlet and maceration, were most widely applied due to their operational simplicity and the preservation of structurally diverse metabolites while percolation, decoction, infusion, and hydro-distillation were sparsely utilized. Extraction strategy and solvent polarity emerged as primary factors shaping phytochemical profiles, with phenolics, flavonoids, alkaloids, and terpenoids identified as dominant classes. Reported half maximal inhibitory concentration (IC_50_) ranged from highly potent (0.12 µg/mL) to weak (30,000 µg/mL), reflecting variability driven by extraction parameters and plant matrix complexity. Anticancer mechanisms commonly involved apoptosis induction, cell-cycle arrest, reactive oxygen species-mediated cytotoxicity, and inhibition of proliferative signaling pathways across breast, cervical, colon, lung, liver, and prostate cancer models. Although solvent-based extraction approaches remain widely used, their context-dependent nature and lack of standardization limit reproducibility. Overall, anticancer and chemotherapeutic efficacy is primarily governed by BAC composition, while extraction methods act as upstream modulators. Future progress requires phytochemical-informed, standardized workflows supported by hybrid extraction systems, AI-assisted optimization, and advanced bioavailability and delivery systems to enable reproducible and clinically relevant translation of plant-derived chemotherapeutics.

## 1. Introduction

Cancer remains one of the most pressing global health challenges and is currently the second leading cause of mortality worldwide after cardiovascular diseases [1,2,3,4,5,6], accounting for ~19 million new cases and 10 million deaths in 2020 alone [7]. Epidemiological projections indicate that the global incidence may exceed 30 million new cases annually by 2040, underscoring the urgent need for innovative therapeutic and preventive strategies. Despite advances in surgery, chemotherapy, targeted therapies (e.g., EGFR and VEGF inhibitors), and immunotherapy, treatment efficacy remains limited for many aggressive and late-stage cancers due to toxicity, drug resistance, and disease recurrence [8]. Multidrug resistance alone contributes to up to 90% of chemotherapy failures, highlighting the need for alternative approaches that improve selectivity while minimizing adverse effects [1,2,3,4,5,6,9,10,11,12]. Plant-derived BACs have gained considerable attention as potential anticancer agents, given their longstanding role in drug discovery. Approximately 68% of small-molecule anticancer drugs developed between 1940 and 2014 were derived from or influenced by natural products [8]. These compounds can induce apoptosis, inhibit proliferation, modulate oxidative stress and inflammatory pathways, and suppress angiogenesis and metastasis, while often displaying lower toxicity toward normal cells [1,2,3,4,5,6,9,10,11,12,13,14]. Compounds such as curcumin, resveratrol, and epigallocatechin gallate exemplify this therapeutic potential. However, their effectiveness depends not only on bioactivity but also on efficient extraction methods that enable their recovery and stability. Plant matrices contain structurally diverse metabolites, often present at low concentrations, making extraction a critical determinant of phytochemical yield and functionality [13,15]. Solvent-based techniques including maceration, percolation, decoction, infusion, Soxhlet extraction, and hydro-distillation remain widely used due to their simplicity and scalability. However, these methods are often associated with long extraction times, high solvent consumption, and degradation of thermolabile compounds [1,2,11,16,17,18,19,20,21,22].

Advanced extraction technologies such as ultrasound-assisted extraction (UAE), microwave-assisted extraction), accelerated solvent extraction, enzyme-assisted extraction, supercritical fluid extraction, pressurized liquid extraction, high hydrostatic pressure extraction, pulsed electric field extraction, and green solvent systems offer significant advantages such as higher extraction efficiencies and yields, shorter extraction durations, reduced use of organic solvents, and better preservation of efficacy of thermolabile metabolites under milder operating conditions [15,23]. By improving the recovery of key phytochemicals, these advanced methods can enhance the potency of plant extracts and ensure that the resulting compounds retain their desired anticancer properties. Despite these advantages, their adoption remains limited due to cost, operational complexity, and technical requirements [15,23]. Consequently, solvent-based extraction methods continue to dominate, even though extraction conditions strongly influence phytochemical composition and bioactivity. The current literature remains fragmented, with substantial variability in reported anticancer activity even for the same plant species. Such inconsistencies arise from differences in extraction parameters, solvent properties, and plant matrix characteristics, which govern the selective recovery of BACs [24,25,26,27]. As a result, there is no clear consensus on optimal extraction strategies for anticancer phytochemicals, limiting reproducibility and comparability across studies. Although several reviews have explored anticancer activities of specific phytochemical classes (e.g., polyphenols or alkaloids) [13], advanced extraction technologies such as ultrasound-assisted or supercritical fluid extraction [15], particular cancer cell models [28], or individual plant species and small plant groups [29,30], few have systematically examined how solvent-based extraction influences phytochemical recovery and downstream anticancer activity across diverse studies.

In this context, the present review synthesizes studies published between 2020 and 2025 that employed solvent-based extraction methods to extract BACs from plants with reported anticancer activity. By integrating extraction parameters with phytochemical composition, IC_50_ variability, and mechanistic endpoints such as apoptosis induction, this review provides a framework linking extraction methodology to phytochemical composition and biological response, highlighting the critical role of extraction in shaping anticancer outcomes.

## 2. Methodology

### Literature Selection and Scope

This review employed a structured literature search to identify studies investigating plant-derived BACs with reported anticancer activity. Electronic databases, including PubMed and Google Scholar, were searched for relevant peer-reviewed articles published between 2020 and 2025. The search strategy combined keywords and Boolean operators such as “solvent extraction,” “plant extract,” “medicinal plants,” “anticancer activity,” “bioactive compounds,” and “cancer cell lines,” which were applied consistently across databases. Retrieved records were screened to remove duplicates prior to evaluation. Study selection was conducted in two stages: initial screening of titles and abstracts, followed by full-text assessment of potentially relevant articles. Eligibility was determined by using predefined inclusion and exclusion criteria. Inclusion criteria comprised: (i) original research articles published in English between 2020 and 2025; (ii) studies reporting extraction of phytochemicals from plant materials; (iii) studies evaluating anticancer activity using in vitro, in vivo, or clinical models; and (iv) articles providing sufficient methodological detail on extraction procedures. Exclusion criteria included review articles, meta-analyses, books, conference abstracts, non-English publications, studies not related to anticancer activity, and articles lacking clear extraction methodology or accessible full texts.

## 3. Results

**Figure 1:**
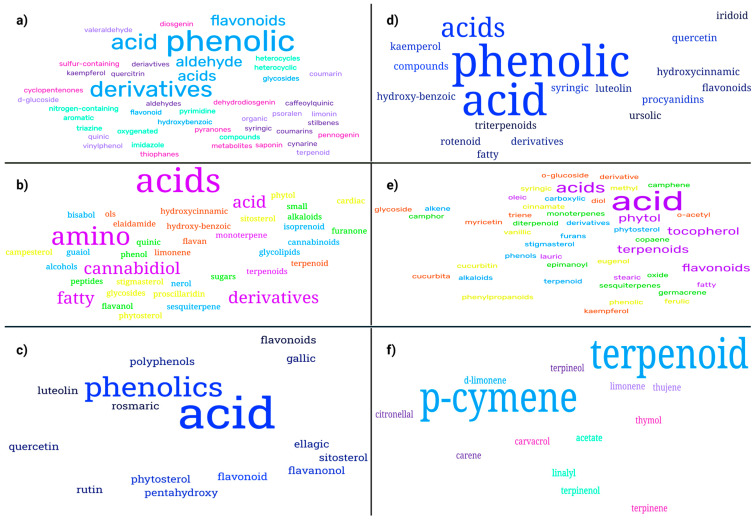
Summary of secondary metabolites linked to anticancer activity identified using (**a**) solvent, (**b**) maceration, (**c**) decoction, (**d**) infusion, (**e**) Soxhlet, and (**f**) hydro-distillation extraction methods.

**Figure 2:**
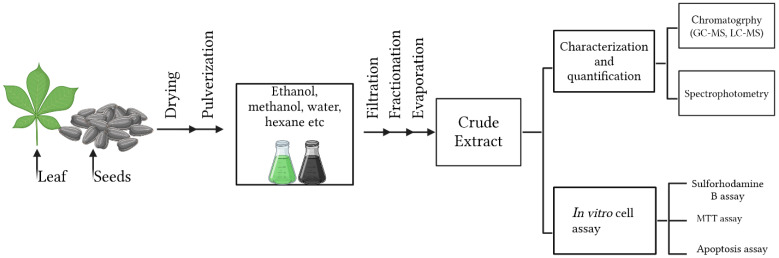
Overview of solvent extraction for isolating phytochemicals and in vitro anticancer assays. Created with BioRender (https://www.biorender.com/).

**Figure 3:**
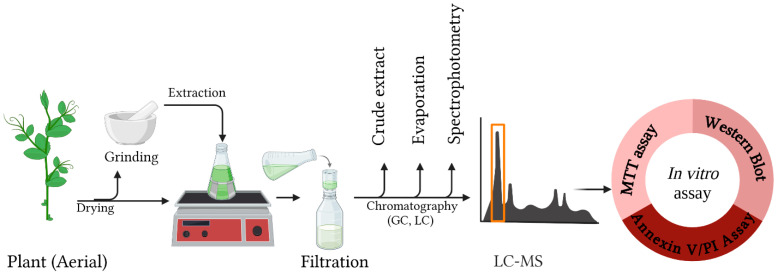
Overview of maceration of plants, characterization of phytochemicals, and in vitro anticancer assays. Created with BioRender.

**Figure 4:**
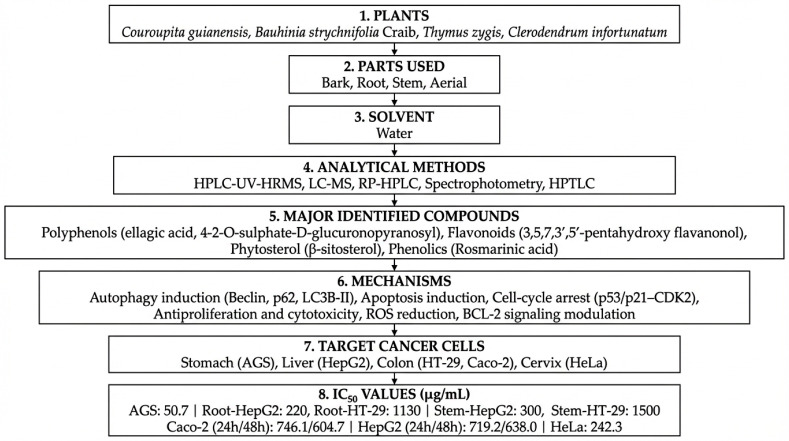
Decoction of plant materials and antiproliferative mechanism. Created with SciSpace Biomedical.

**Figure 5:**
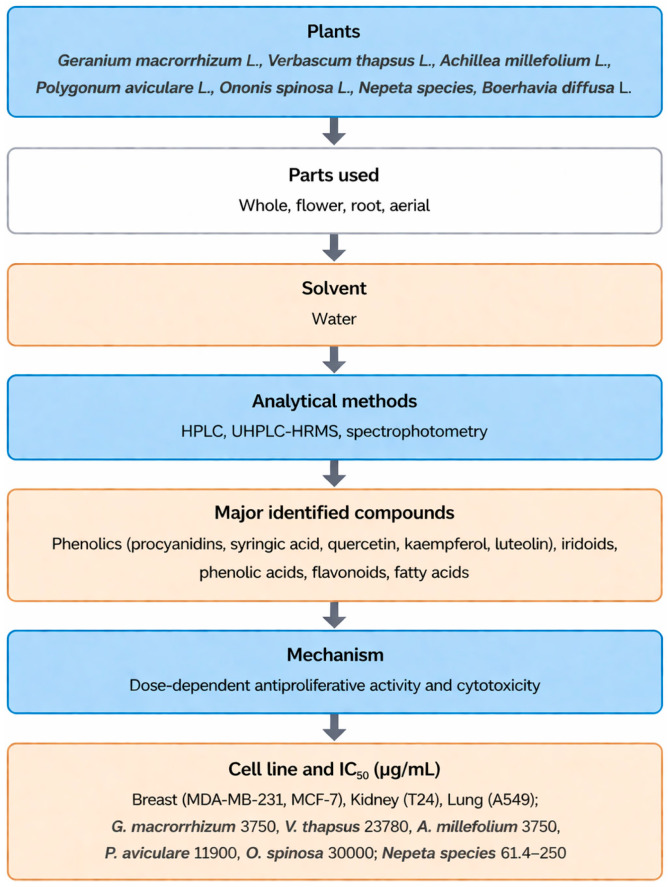
Overview of infusion and antiproliferative mechanism of plant extracts. Created with SciSpace Biomedical.

**Figure 6:**
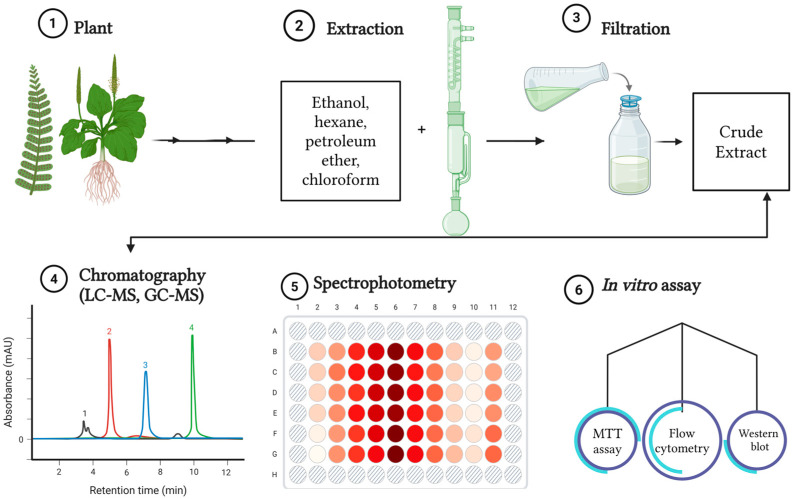
Soxhlet extraction of phytochemicals and in vitro anticancer assays. Created with BioRender.

**Figure 7:**
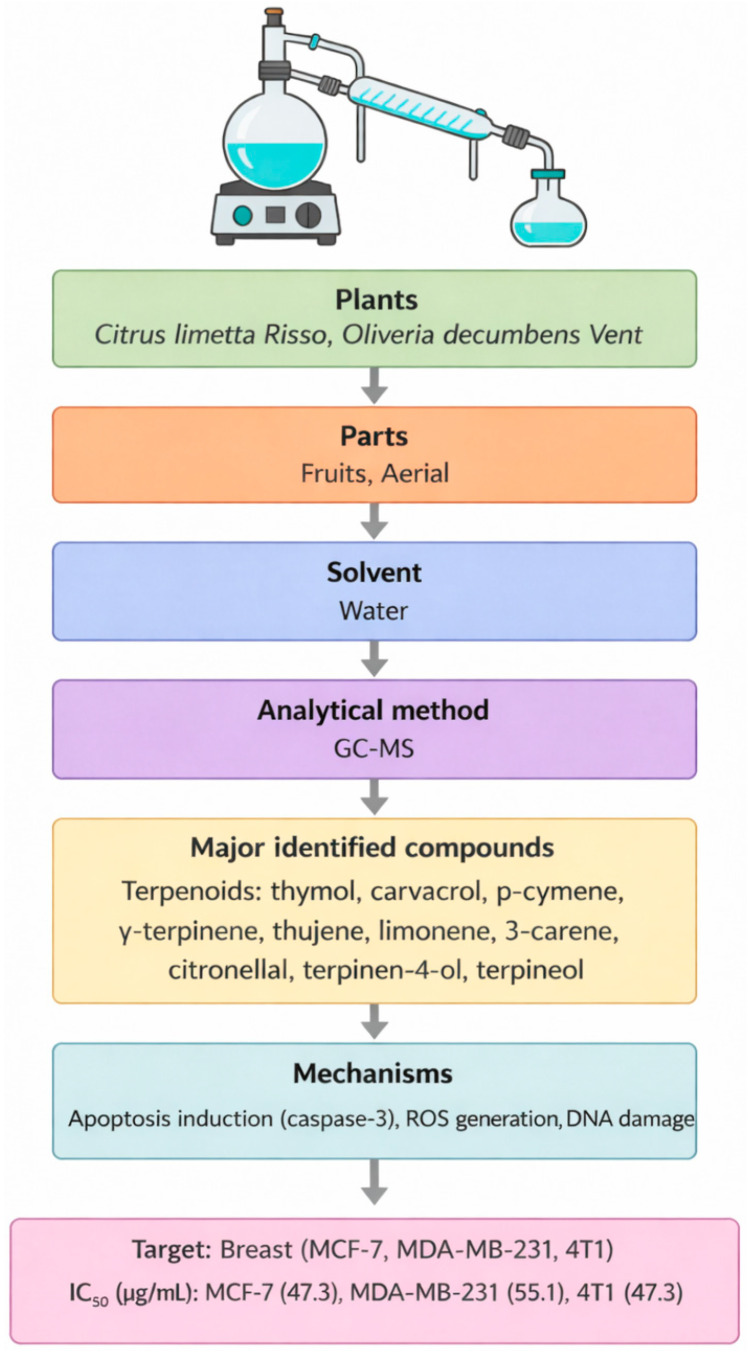
Hydro- and steam distillation and bioactivity of recovered phytoconstituents on selected cancer cell lines. Created with SciSpace Biomedical.

The selected studies investigated a diverse range of medicinal plants and reported detailed information on plant parts used, extraction methods, identified phytochemicals, anticancer mechanisms, IC_50_ values, and the cancer models employed for biological evaluation. Across the studies included, extraction approaches varied considerably, reflecting differences in research objectives, phytochemical targets, and methodological preferences. Solvent-based techniques represented the most frequently employed extraction strategies, particularly maceration, Soxhlet, and general solvent extraction were most frequently employed due to their ability to recover a broad spectrum of phytochemicals and their suitability for laboratory-scale screening. In contrast, traditional methods such as percolation, infusion, and decoction were less commonly applied, likely reflecting their stronger association with ethnomedicinal practice than experimental anticancer research. Differences in extraction techniques were associated with variations in phytochemical profiles and anticancer potency, as evidenced by the wide range of IC_50_ values reported across studies. Most extracts were rich in secondary metabolites, including polyphenols, flavonoids, alkaloids, and terpenoids, which were consistently linked to anticancer activity (Figure 1). These compounds exert their effects through multiple mechanisms, including apoptosis induction, inhibition of cell proliferation, cell-cycle arrest, and oxidative stress-mediated cytotoxicity. Commonly investigated models included breast, colon, lung, cervical, prostate, and liver cancer cell lines, although the magnitude of cytotoxic effects varied depending on extraction conditions, phytochemical composition, and experimental design. Table 1, Table 2 and Table 3 summarize the studies reviewed under selected extraction methods, detailing the plant species investigated, extraction procedures, identified phytochemicals, cancer cell models, reported IC_50_ values, and the associated anticancer mechanisms. In addition, Figure 2, Figure 3, Figure 4, Figure 5, Figure 6 and Figure 7 present a conceptual overview summarizing the different extraction methods examined.

### 3.1. Influence of Pre-Extraction Processing and Extraction Optimization

The recovery of BACs from plant matrices is shaped not only by solvent composition, extraction time, and temperature, but also by upstream processing steps that define the chemical and structural state of the material before extraction begins. Variability in pre-extraction treatment and extraction design is therefore a major, yet often underreported, source of inconsistency across phytochemical studies. Since plant matrices contain metabolites that differ in polarity, stability, localization, and association with structural components, extraction outcomes depend as much on matrix preparation and extraction completeness as on solvent choice itself [1,2,3,4,9,11,19,31,32,33,34,35,36,37,38,39,40,41,42,43,44]. Among upstream variables, drying and size reduction are especially important as they affect both compound stability and solvent accessibility. Across the reviewed studies, shade drying at ambient temperature was used more frequently than oven drying under controlled thermal conditions [1,2,3,4,9,11,19,31,32,33,34,35,36,37,38,39,40,41,42,43,44], whereas oven drying was applied in a smaller group [18,45,46,47,48]. These approaches are not functionally equivalent.

Shade drying generally preserves thermolabile metabolites more effectively, whereas oven drying can accelerate oxidative and thermal degradation of compounds such as flavonoids, phenolic acids, and volatile constituents [18,45,46,47,48]. Although oven drying improves processing speed, it may compromise phytochemical integrity, while shade drying better preserves compounds likely to contribute to the observed anticancer activity. Drying also reduces moisture content, thereby improving solvent penetration and limiting enzymatic degradation, but differences in drying method and particle size can still alter extraction efficiency even when the same solvent system is used. This indicates that variation in pre extraction handling can influence both phytochemical yield and bioactivity independently of solvent selection. Extraction design introduces an additional layer of variability. Studies using repeated extraction cycles with fresh solvent generally reported more complete metabolite recovery than single step approaches, although the extent of improvement depended on plant matrix structure and compound localization [18,39,48,49,50,51,52]. This pattern reflects the structural constraints of plant tissues, where cellulose, hemicellulose, and lignin can limit solvent access to intracellular metabolites. Repeated extraction therefore improves recovery not simply by increasing solvent exposure, but by progressively releasing compounds that remain partially retained after the initial extraction [18,39,48,49,50,51,52]. Sequential extraction extends this principle by targeting different phytochemical fractions under different conditions. Compared with single step extraction, sequential strategies often produced broader phytochemical profiles and revealed metabolites that would otherwise remain underrepresented, including bound phenolics associated with cell wall structures [53]. In several studies, inclusion of these less accessible fractions was associated with enhanced antioxidant or cytotoxic activity, indicating that extraction completeness can substantially alter the biological profile attributed to the extract [53].

Systematic optimization approaches further distinguish controlled extraction design from empirical practice. Statistical tools such as response surface methodology, often combined with Box–Behnken design, allow simultaneous evaluation of interactions among solvent composition, extraction time, temperature, and solid to solvent ratio [54]. By moving beyond trial and error, these approaches provide a predictive framework for maximizing phytochemical recovery and improving reproducibility. Studies that applied such optimization strategies reported phenolic yields closely aligned with predicted values, whereas non optimized extraction procedures often showed greater variability in phytochemical composition and bioactivity [54]. This contrast indicates that poor comparability across studies is driven not only by biological differences in plant material, but also by inconsistent control of interacting extraction variables. Overall, the reviewed literature shows that extraction outcomes are governed by an interplay of pre-extraction processing, plant matrix architecture, and extraction design. These factors do not merely influence yield; they determine which metabolites are preserved, released, or underrepresented before biological testing is performed. As a result, differences in reported anticancer activity across studies cannot be attributed to plant species or solvent system alone but must also be interpreted considering drying method, extraction completeness, and optimization strategy. Greater emphasis on systematic optimization and transparent reporting of upstream processing conditions will therefore be essential for improving reproducibility, strengthening cross-study comparison, and linking phytochemical composition more reliably to bioactivity in anticancer research.

### 3.2. Extraction of Plant-Derived Natural Products

#### 3.2.1. Solvent Extraction

Solvent extraction remains a central strategy in phytochemical research for alternative cancer therapeutics not simply owing to it is convenience, but as it provides a controllable chemical interface between a complex biological matrix and the classes of metabolites targeted for extraction. Encompassing both simple approaches such as maceration and more intensive methods including Soxhlet extraction [15,23,55,56,57,58], its continued use in phytochemical studies reflects more than methodological flexibility, scalability, but underscores that solvent systems can be tuned to favor the release of metabolites with markedly different physicochemical properties, biological reactivities, and mechanistic roles in cancer inhibition while preserving thermolabile constituents that may undergo degradation under more aggressive processing conditions [19,42,59]. A schematic overview of solvent extraction is presented in Figure 2.

##### Influence of Solvent Polarity and Plant Part on Extraction

Extraction efficiency and anticancer profile of plant-derived extracts are determined by the combined influence of solvent polarity and plant part selection. Although these variables are often discussed independently, the reviewed literature indicates that they operate as interdependent determinants of both phytochemical recovery and biological response. Solvent polarity governs which metabolites can be solubilized, whereas plants part determines which metabolites are present and available for extraction. This distinction is central to cross-study interpretation since differences in extract composition and anticancer potency cannot be attributed to solvent choice alone when the underlying plant matrix also differs [1,3,4,5,6,10,12,21,31,32,43,44,45,50,51,52,53,59,60,61,62,63,64,65,66,67,68,69,70].

Across the literature, polar solvents such as methanol, ethanol, and water generally favored the recovery of hydrophilic metabolites, including phenolics, flavonoids, glycosides, alkaloids, and related phenolic derivatives, whereas less polar solvents more often enriched lipophilic constituents such as terpenoids, sterols, and other nonpolar metabolites. Intermediate polarity systems, particularly hydro-alcoholic mixtures, broaden the extraction window by recovering both polar and moderately lipophilic compounds [6,12,43,45,66,69]. However, broader recovery does not consistently correspond to stronger anticancer activity. Instead, the reviewed studies indicate that chemically broader extracts often improve metabolite coverage without necessarily improving selective enrichment of the constituents most responsible for cytotoxic or antiproliferative effects (Table 1) [3,5], [21,31,51,52,60,68]. In this context, extraction breadth should not be interpreted as equivalent to biological potency.

A similar pattern is evident in multi-solvent and sequential extraction workflows [3,5,10,21,31,32,51,52,59,60,63,64,65,68]. This distinction is important as it shows that maximizing extraction yield and achieving selective enrichment are related but fundamentally different objectives. Accordingly, variability in anticancer response across extracts may reflect differences in chemical resolution rather than simple differences in recovery efficiency. Plant part selection introduces an equally important and often greater source of variability. Different plant organs accumulate distinct metabolite profiles according to their physiological roles, with aerial tissues more frequently associated with soluble phenolics and flavonoids, and roots, bark, rhizomes, and seeds more often associated with structurally bound or relatively lipophilic constituents [1,12,42,45,65]. As a result, extracts obtained under similar solvent conditions can still differ markedly in composition and bioactivity when derived from different plant parts. This source of variability is further amplified by developmental stage, geographic origin, climate, harvest timing, and post-harvest handling, all of which influence metabolite abundance before extraction begins. When considered collectively, the reviewed literature supports a hierarchical interpretation of extraction variability. Plant material defines the available chemical space, whereas solvent polarity determines which portion of that space is recovered. Within this framework, solvent optimization can improve extraction efficiency only within the limits imposed by the starting matrix, but it cannot compensate for major differences in tissue specific composition, developmental stage, or source related variation. It also clarifies why extraction efficiency shouldn’t be judged by yield. Therefore, solvent polarity and plant parts should be treated as interdependent variables in comparative analysis, and differences in bioactivity should not be attributed to extraction method alone without accounting for underlying variation in plant material.

**Table 1:** Composition, bioactivity and anticancer potential of solvent-extracted phytochemicals.

Plant	Part (s) Used	Solvents	Analytical Method	Major Identified Compounds	Mechanisms	Target Organ/Cell Line and Assay	IC_50_ (µg/mL)	References
*Eruca sativa*	Leaf	Ethanol	HR-LC/MS	Vitamins, alkaloids, fatty acids, flavonoids, terpenoids and phenols	Reduced cell viability	Colon (HCT-116, Caco-2), MTT assay	HCT-116 (64.9) and Cac0-2 (84.0)	[45]
*Adenosma bracteosum*	Aerial	Ethanol	NMR	5,4′-dihydroxy-6,7,8,3′-tetramethoxyflavone (AB2), ursolic acid	Apoptosis induction (Caspase-3 activation) and ROS-mediated cytotoxicity	Liver (NCI-H460) and lung (HepG2), Sulforhodamine B (SRB) assay	AB2: NCI-H460 (4.6) and HepG2 (5.7), and ursolic acid: NCI-H460 (13.1) and HepG2 (10.0)	[42]
*Allium tuncelianum*	Aerial	Methanol	LC-MS/MS	Phenolic acids (hydroxybenzoic acids), flavonoids, and flavonoid glycosides (quercitrin), stilbenes, coumarins, phenolic aldehydes (syringic aldehyde), and caffeoylquinic acid derivatives (cynarine)	MAT increased Caspase-3 and Caspase-9 expression and suppressed IL-6, IL-8, MMP-2, and MMP-9 expression	Prostate (PC-3), ECC-1 colon (DLD-1), cervix (HeLa), Kidney (HEK-293)	PC-3 (105.7), DLD-1 (111.7), ECC-1 (105.9), HeLa (134.4), HGC-27 (180.3), and HEK-293 (185.4)	[6]
*Azadirachta indica*	Seed	Methanol	GC-MS	Flavonoids (kaempferol deriavtives), aromatic phenolic derivatives, nitrogen-containing heterocyclic compounds (pyrimidine, imidazole, and triazine derivatives), oxygenated heterocycles (pyranones and cyclopentenones), and sulfur-containing metabolites (thiophanes)	Apoptosis induction, Cell proliferation inhibition, reduced CD44 and CD326 population	Breast (MDA-MB-231), MTT assay	Ripe neem seed: MDA-MB-231 (10) and unripe neem seed: MDA-MB-231 (30)	[43]
*Excoecaria agallocha*	Leaf	Water	HR LC-MS	Phenolics, phenolic acid, terpenoids, azole derivatives	Autophagy and mitophagy apoptosis induction, (SMAC-activation) cell-cycle arrest (G2/M) p53/p21–cyclin pathway	Cervix (SiHa HPV 16+), MTT assay	SiHa HPV (15.5)	[12]
*Withania somnifera*	Leaf	Water	Spectrophotometry	Phenolics, flavonoids, tannins	Cell growth inhibition	Cervix (HeLa), larynx (HEp-2), prostate (PC3), breast (MCF-7), colon (HCT-116), liver (Hep-G2) lung (WI-38) MTT assay	Hep-G2 (35.7), MCF-7 (52.8), HCT-116 (57.5), PC3 (61.2), HeLa (32.1), Hep 2 (43.1), and WI-38 (92.4)	[66]
*Helichrysum* *arenarium*	Aerial flower	Ethanol, methanol	LC-MS-QTOF	Flavonoids (naringenin, luteonin), flavonoid glycosides (aureusidin-6-rhamnoside), terpenoids (16-deoxomexicanolide), phenolic derivatives (gmelinol, athamantin)	Cytotoxicity	Bladder (RT-4), brain (T98G) and breast (MDA-MB-231), MTS assay	Ethanol (24 & 48 h): T98G (NA, 1.9), MDA-MB-231 (0.2, 0.1), RT4 (1.8, 1.1), methanol: 24 and 48 h, T98G (NA, 2.1), MDA-MB-231 (1.3, 0.2), RT4 (NA, 1.8)	[61]
*Anacardium occidentale* L.	Leaf	Hydro-ethanol	LC-MS	Pentagalloylglucose	ROS-mediated oxidative cytotoxicity	Cervix (HeLa) and lung (MRC5-SV2)	Cervix (71.5), and lung (52.2)	[1]
*Prangos pabularia*	Roots	Hydro-ethanol	Not reported	Not reported	Early and late apoptosis and necrosis	Breast (MCF7), MTT assay	24 h (756), 48 h (682), and 72 h (450)	[3]
*Tabernaemontana catharinensis*	Stem	Hydro-ethanol	UPLC-Q-TOF/MS and HPLC	Phenolic acids (caffeic acid, protocatechuic acid, chlorogenic acid) phenolic glycosides (protocatechuic acid 4-glucoside), phenylpropanoids (coniferyl aldehyde)	Apoptosis induction (caspase-3/7, -8, -9), mitochondrial dysfunction, p53 upregulation and G1-phase cell-cycle arrest	Breast (MCF-7 and MDA-MB-231)	MCF-7: 83.1 and 499.3; MDA-MB-231: 8.3 and 280.0	[62]
*Paris polyphylla*	Rhizome	Hydro-ethanol	GC-MS, HPTLC	Saponin (diosgenin, pennogenin, 7β-dehydrodiosgenin)	Apoptosis induction (Bcl-2 family proteins (Bcl-2 downregulation, Bax upregulation) and survivin suppression)	Breast (MCF-7, MDA-MB-231), cervical (HeLa) (Hep-2)	24, 48 and 72 h MDA-MB-231 (101.6, 65.8, 40.8), MCF-7 (34.4 23.8 and 20.2), HeLa (97.4, 68.7, 47.9), and Hep-2 (55.2, 34.0, and 26.7)	[44]
*Zygophyllum coccineum* L.	Whole	Hydro-ethanol	LC-ESI-TOF-MS, Spectrophotometry	Alkaloids (spermine spermidine, kynurenic acid), stilbenes (isorhapontin, abscisic acid, gibberellin-A4), flavonoids (kaempferol-3-O-(p-coumaroyl)-glucoside), saponins (zygophyloside-F, zygophyloside-G)	Topoisomerase IIβ inhibition, DNA damage, and apoptosis induction	Breast (MCF-7), colon (HCT-116), and liver (HepG2)	MCF-7 (3.5), HCT-116 (3.2) and HepG2 (2.3)	[4]
*Eleusine coracana*	Seeds	Hydro-ethanol	UPLC-QTOF-MS	Phenolic acids (hydroxybenzoic acids: vanillic acid and protocatechuic acid), hydroxycinnamic acids (ferulic acid)	G0/G1 or G2/M cell-cycle arrest (DNA fragmentation and Sub-G1 apoptotic accumulation)	Colon (HCT-15, HCT-116, HT-29) breast (MCF-7, MDA-MB-231, and MDA-MB-468)	N/A	[53]
*W. somnifera* and *Arctium lappa*	Aerial	Hydro-ethanol	Spectrophotometry	Phenolics, flavonoids	G2/M cell cycle arrest, apoptosis, necrosis	Breast (MCF-7), MTT assay	MCF-7: WS (47.1) and AL (149.8)	[70]
*Gnetum montanum*	Stem and rhizome	Hydro-ethanol	HPLC-Q-TOF-MS/MS	Stilbenes (resveratrol, gnetumontanin c), flavonoids (naringenin), sterols, and alkaloids, phenolic acids (vanillic acid)	G2/M cell-cycle arrest, apoptosis induction (Caspase-3), AKT pathway inhibition	Colon (SW480)	SW480 (24, 48, 72 h): 126.5, 78.3, and 50.8	[50]
*Atriplex halimus* L.	Leaf	Hydro-ethanol	LC-MS/MS	Phenolic acids (gallic acid, syringic acid, trans-ferulic acid, caffeic acid, chlorogenic acid), flavonoids (myricetin, catechin gallate)	Antiproliferative and cytotoxic activity	Breast (MCF-7 and MDA-MB-231), MTT assay	MCF-7 (27.9) and MDA-MB-231 (52.0)	[31]
*Selaginella repanda*	Whole	Hydro-ethanol	Spectrophotometry, HR-LC–MS	Terpenoids (citral, pulegone), coumarins (7-hydroxycoumarin), amino acids, fatty acids (arachidonic acid), sugars and sugar alcohol	Antiproliferation and apoptosis induction	Lung (A549), colon (HCT-116), and breast (MCF-7)	Lung (A549) (341.1) colon (HCT-116) (378.8), and breast (MCF-7) (428.3)	[60]
*Thymus zygis*	Aerial	Hydro-ethanol	RP-HPLC, spectrophotometry	Flavonoid glycosides (apigenin-(6,8)-C-diglucoside), polyphenolic acids, triterpenoids	Antiproliferation and cytotoxicity	Colon (Caco-2) and Liver (HepG2)	Hydro-ethanol: Caco-2 (24, 48 h): 32, 85.0 and HepG2 (24, 48 h) (120, 82.2)	[52]
*Salix safsaf*	Leaf	Hydro-ethanol	UHPLC-QHFMS	Phenolic, flavonoids (aglycones and glycosides), iridoids, and purine/cytokinin derivatives	Ethyl acetate and chloroform fractions exhibited apoptosis, ROS-generation	Breast (MCF-7), colorectal (HCT-116), cervical (HeLa) and liver (HepG2), MTT assay	Ethyl acetate: MCF-7 (111.7), HCT-116 (195.6), HeLa (156.2), HepG2 (172.39), chloroform: MCF-7 (128.1), HCT-116 (151.5), HeLa (141.6) and HepG2 (136.7)	[71]
*Anisomeles indica*	Aerial	Hydro-ethanol	HPLC-MS	Ovatodiolide	G2/M cell-cycle arrest (Bax upregulation, Bcl-2 downregulation), apoptosis induction	Stomach (AGS)	AGS (24 and 48 h): 4.3 and 2.0	[67]
*Drimia calcarata*	Stem	Methanol and water	LC-MS	Flavonoid (eriodictyol-7-O-glucoside), phenolic acid (chlorogenic acid), terpenoid (limonin-17-D-glucoside) and coumarin (psoralen)	Bcl-2 downregulation (STAT3/STAT5B suppression), Apoptosis induction	Lung (H1573, A549 and H1437), MTT assay and Annexin V	Methanol: H1573 (125), and H1437 (125), A549 (500) Water: H1573 (125) and H1437 (250)	[32]
*Cuscuta epithymum*, *Achillea millefolium*, *Salvia officinalis*, *Salvia hydrangea*, and *Teucrium polium*	Aerial	Hydro-methanol	GC-MS	Organic acid (quinic acid), phenolic (vinylphenol), aldehyde (valeraldehyde)	STAT4/PIK3CD/EMP1↑ and RAB11FIP3↓ gene expression modulation	Pancreas (MIA PaCa-2 and PaTu 8902)	*C. epithymum*: MIA PaCa-2 (85.0) and PaTu 8902 (156.6), *A. millefolium:* MIA PaCa-2 (338.40) and PaTu 8902 (343.69), *S. hydrangea:* MIA PaCa-2 (569.0) and PaTu 8902 (270.1), *S. officinalis:* MIA PaCa-2 (458.2) and PaTu 8902 (336.9) and *T. polium:* MIA PaCa-2 (205.6) and PaTu 8902 (421.8)	[5]
*Ginkgo biloba* L.	Seed	Hydro-methanol	LC-MS/MS	Terpenoid (ginkgolide a. ginkgolide b), flavonoid (quercetin, kaempferol)	Induced cell death	Skin (A2058) and colon (HCT116)	A2058 (330) and HCT-116 (455)	[51]
*Juniperus sabina* and *Ferula communis*	Leaf	Hydro-methanol and hydro-ethanol	GC-MS, Spectrophotometry	Terpenoids (sesquiterpene, diterpene), phenols, esters	Antiproliferation and cytotoxicity	Breast (MCF-7, MDA-MB-231), MTT assay	MCF-7 (61.3) and MDA-MB-231 (18.2)	[68]
*Quercus coccifera*	Leaf	Methanol, chloroform, hexane, and water	Spectrophotometry	Alkaloids, tannins, terpenoids, glycosides	Cytotoxicity	Lung (NCI-H2126), breast (BT-20), and prostate (DU-145)	Methanol (72.5, 27.4, and 53.2), boiled water (14.1, 7.2, and 25.1) and microwaved water (117.8, 39.6, and 57.3)	[65]
*Suaeda maritima*	Leaf	Hexane, chloroform methanol, and water	LC-MS/MS, spectrophotometry	Phenolics, flavonoids (formononetin, 7-O-beta-glucopyranosyl-4-hydroxy-5-methoxyisoflavone), steroids, and triterpenoid derivatives	Apoptosis induction	Lung (A549), MTT assay	52.6	[63]
*Ceratonia siliqua* L.	Seeds, pulp and whole	Diethyl ether, ethyl acetate, ethanol, and water	LC-MS, spectrophotometry	Flavonoids (naringenin, kaemferol), phenolic acids (ferulic acid, gallic acid)	Apoptosis induction (Caspase activation)	Breast (MCF-7, MCF-10A), MTT assay	Pulp + seeds, MCF-7 (440, 260, 760, and 2180), MCF-10A (5430, 5740), Pulp: MCF-7 (270, 960), MCF-10A (2340), Seeds: MCF-7 (260), MCF-10A (570), whole (pulp + seeds): MCF-7 (820, 420), MCF-10A (1750), pulp: MCF-7 (1450, 280), and MCF-10A (290, 380)	[64]
*Nerium odorum*	Leaf	Ethanol, methanol, chloroform, ethyl acetate, and water	GC-MS, FT-IR, HPTLC, spectrophotometry	Furan derivatives (5-hydroxymethylfurfural), lipid derivatives (2,3-Bis[(trimethylsilyl)oxy]propyl ester) hydrocarbons (Octadecane), carbohydrate compounds (β-D-allopyranoside derivatives) and cyclic ketones.	Induce cytotoxicity	Breast (MDA-MB 231), colon (HT-29) WST-1 assay	Ethyl acetate (2200)	[59]
Globe artichoke	Bracts and receptacles	Hexane, diethyl ether anhydrous, ethanol and double-distilled water	HPLC, spectrophotometry	Phenolics (protocatechuic, vanillic, chlorogenic, ferulic), flavonoids (apigenin-7-glucoside)	Cytotoxicity	Liver (HepG2), breast (MCF7), MTT assay	Receptacles: HepG2 (661) and MCF-7 (1724), Bracts: HepG2 (514), and MCF-7 (847)	[10]

##### Fractionation and Purification of Bioactive Compounds

Following extraction, fractionation is widely used to resolve crude plant extracts into simpler chemical fractions, thereby linking extract level bioactivity to the metabolites responsible for the observed anticancer effects [1,4,42,64]. Since crude extracts contain complex mixtures of phytochemicals, bioactivity measured at the whole extract level cannot be assigned confidently to individual constituents without further separation and analytical characterization. In the reviewed literature, the extent of fractionation differed substantially, and this methodological difference strongly influenced how anticancer activity could be interpreted. Studies that incorporated progressive fractionation together with structural characterization generally provided clearer mechanistic attribution, including identification of compounds associated with apoptosis induction, caspase activation, and cell cycle arrest [1,4,42,64]. In contrast, studies that are limited to crude extract evaluation more often reported cytotoxic or antiproliferative effects without clearly identifying the responsible constituents. This comparison indicates that studies with and without fractionation do not represent equivalent levels of evidence. This distinction also helps explain variability in reported potency. Crude extracts may appear less active or more mechanistically ambiguous, not necessarily because they lack relevant BACs, but since active constituents remain diluted within chemically complex mixtures. By contrast, fractionated samples often display stronger or more clearly defined effects as enrichment increases the relative abundance of the metabolites most closely associated with anticancer activity. This is a major limitation in cross-study comparison, since similarity in extraction method does not ensure biological equivalence when the degree of downstream chemical resolution differs.

At the same time, fractionation should not be interpreted as an unqualified improvement in all cases. Although purification enhances mechanistic clarity, it can also reduce the biological complexity present in crude extracts, where multiple co-occurring metabolites may act additively or synergistically. Consequently, isolated compounds may provide stronger causal evidence for specific mechanisms, while crude extracts may better reflect the combined biological behavior of plant matrices. Beyond mechanistic clarification, fractionation also functions as a prioritization strategy within phytochemical anticancer research. By identifying the fractions or compounds most strongly associated with bioactivity, it helps determine which plant species, plant parts, and extraction conditions warrant further structural elucidation, mechanistic validation, and preclinical evaluation [1,4,42]. However, literature also highlights an important translational constraint. Chromatographic fractionation is effective at laboratory scale but scaling compound isolation for larger preclinical or industrial applications remains difficult [67]. In this context, centrifugal partition chromatography has emerged as a promising alternative since it reduces irreversible adsorption and sample loss during purification [67]. Even so, its broader application depends on optimization of biphasic solvent systems, automation, and solvent recycling to improve economic feasibility. Overall, the reviewed studies show that fractionation is not merely a downstream analytical step, but a determinant of how anticancer activity is resolved, interpreted, and compared across the literature. Its inclusion strengthens mechanistic attribution and can increase apparent activity through compound enrichment, whereas its absence may obscure the contribution of key bioactive constituents. This indicates that variability in reported anticancer effects is shaped not only by plant chemistry, but also by differences in analytical strategy. 

##### Variability in IC_50_ Values Across Cancer Cell Models

According to the United States National Cancer Institute (NCI), crude extracts are generally considered to show significant in vitro cytotoxic activity when the IC_50_ value is ≤30 µg/mL [72]. However, the reviewed literature reports a very wide IC_50_ range, from 0.1 to 5740 µg/mL, even among studies using comparable extraction approaches (Table 1) [1,3,4,5,6,10,12,21,31,32,43,44,45,50,51,52,53,59,60,61,62,63,64,65,66,67,68,69,70]. This variation indicates that IC_50_ should not be interpreted as a fixed intrinsic property of a plant extract, but rather as a context-dependent measure shaped by multiple interacting factors. At the chemical level, phytochemical composition remains the primary determinant of intrinsic cytotoxic potential. Comparative analysis of the literature shows that extracts prepared using similar solvents or broadly comparable extraction conditions can still produce markedly different IC_50_ values, since they differ in abundance and interaction of bioactive constituents [3,4,31,44,60,62,70]. Yet chemistry alone does not fully explain the spread in reported potency. Differences in effective cellular exposure add a second layer of variability. Although cytotoxicity is usually expressed as crude extract concentration in µg/mL, this measure does not reflect the actual intracellular dose of active compounds delivered to cells. Across studies, variation in stock solution preparation, solvent vehicles, dilution strategies, concentration ranges, and dose response resolution means that nominally similar extract concentrations may correspond to substantially different levels of bioactive exposure. As a result, apparent differences in IC_50_ may arise not only from extract chemistry, but also from how exposure is experimentally defined. This point is especially important in cross-study interpretation, as similar reported concentrations do not necessarily represent equivalent biologically active doses. Biological context further modulates how intrinsic extract activity is expressed. Cancer cell lines differ in genetic background, metabolic capacity, membrane properties, proliferative behavior, redox regulation, and susceptibility to apoptosis or cell cycle disruption. Accordingly, the same extract may produce different IC_50_ values across cancer models as its dominant metabolites may align differently with the vulnerabilities of each system. In this sense, variation in IC_50_ across cell lines reflect not only differences in overall sensitivity but also differences in pathways including responses linked to oxidative stress, mitochondrial dysfunction, cell cycle control, or apoptotic signaling. Methodological factors introduce an additional source of variation. Differences in exposure duration, viability assay, and experimental conditions can systematically alter measured IC_50_ values. Longer incubation periods may yield lower apparent IC_50_ values as some antiproliferative and pro-death effects require time to develop. Under these conditions, shorter exposures can underestimate the activity of extracts with delayed biological responses [3,44,50,52,61,67]. Assay choice can also influence apparent potency as different endpoints capture different aspects of cell injury and viability. Consequently, divergent IC_50_ values may arise in at least four comparison contexts: different extracts tested in the same cell model, the same extract tested across different cell models, similar extraction systems applied to different plant species, and comparable chemical systems evaluated under different assay conditions. These comparison contexts are not equivalent and should not be interpreted as if they reflect the same source of variation.

Collectively, the reviewed literature shows that IC_50_ variability arises from an interdependent system in which extract composition, effective dosing, cell line, and assay jointly shape the observed response. This explains why similar extraction approaches can still produce different biological outcome. It also clarifies that IC_50_ should not be treated as an absolute indicator of extract potency, but as a context dependent measure of expressed cytotoxicity. Therefore, meaningful cross-study comparison requires closer integration of characterization with biological evaluation, clearer reporting of dosing strategies and assay conditions, and greater standardization in experimental design to improve the reliability and interpretability of phytochemical anticancer research.

#### 3.2.2. Maceration

Maceration is a solvent extraction method in which plant material is immersed in a solvent under ambient conditions for a defined period to allow passive diffusion of intracellular metabolites into the extraction medium [2,11,47,73,74,75] (Figure 3). Its continued use in phytochemical anticancer research reflects two major advantages: operational simplicity and the ability to preserve thermolabile constituents in the absence of elevated temperature. However, unlike extraction methods that use heat, pressure, or mechanical intensification, maceration is fundamentally diffusion dependent. As a result, its performance is determined not only by solvent choice, but also by solvent penetration, plant tissue accessibility, extraction duration, and the extent to which target metabolites can diffuse from the plant matrix into the surrounding solvent. Across the literature, these factors collectively shape both the chemical composition of macerates, and the bioactivity later attributed to them. Maceration should therefore be interpreted not simply as a low technology extraction technique, but as a low intensity extraction system in which variability in phytochemical recovery arises primarily from mass transfer limitations and plant matrix effects.

##### Matrix Accessibility and Solvent Dependent Recovery in Maceration

Under maceration conditions, variability in phytochemical recovery is shaped less by solvent polarity alone than by the interaction between solvent dependent solubility and matrix accessibility. Recovery depends not only on whether a metabolite is chemically soluble in the selected solvent, but also on whether that metabolite can diffuse effectively from the plant tissue into the surrounding medium. In this sense, maceration amplifies the influence of plant matrix structure on extraction performance. Across maceration studies, solvent choice still influences the classes of compounds recovered, but the extraction outcome cannot be interpreted based on solvent class alone. Similar solvent systems can produce different phytochemical profiles and anticancer responses when the underlying plant tissues differ in structural organization, metabolite localization, developmental stage, or post-harvest condition. This indicates that, under maceration, solvent selectivity operates within the limits imposed by tissue level accessibility. As a result, extraction differences often reflect how readily metabolites are released from the plant matrix rather than solvent chemistry alone [2,9,11,18,19,39,40,41,47,48,54,73,74,75,76,77,78,79,80,81,82,83,84,85,86,87,88,89,90,91,92] (Table 2). This interaction also helps explain why maceration derived extracts frequently behave as chemically broad and variably selective mixtures. Since passive soaking does not strongly discriminate among compounds beyond solubility and diffusibility, the resulting extracts may contain multiple co-extracted constituents whose collective activity is not easily attributed to a single dominant metabolite. Accordingly, differences in anticancer activity among macerates should be interpreted as the outcome of solvent dependent recovery acting through matrix constrained diffusion, rather than as a direct readout of solvent polarity alone. Collectively, the maceration literature shows that solvent choice remains important, but its effect is mediated by plant matrix accessibility [2,9,11,41,47,69,75,87,91]. 

##### Influence of Extraction Duration

In maceration, extraction duration should not be interpreted as a variable that uniformly increases phytochemical recovery and anticancer efficacy. Since the method is diffusion dependent, duration acts selectively by determining which metabolites are recovered, in what proportions, and at what stage of solvent contact. Time therefore does not simply increase extraction output, but progressively reshapes extract composition and, consequently, the bioactivity associated with the final macerate. Across the reviewed literature, short maceration periods generally favor the recovery of readily soluble metabolites, particularly phenolic acids, flavonoids, and other relatively accessible hydrophilic constituents, whereas intermediate durations allow greater solvent penetration and broader recovery of moderately accessible compounds [9,18,19,48,81,85,87]. This helps explain why intermediate extraction periods are frequently adopted in practice, as they often improve phytochemical coverage while maintaining a relatively high proportion of bioactive constituents [2,22,40,41,47,48,86,91,92]. However, extending maceration beyond this range does not consistently enhance anticancer activity. Although prolonged extraction often increases total yield, it also promotes co extraction of less active or inactive constituents, thereby reducing the relative abundance of the metabolites most responsible for cytotoxic or antiproliferative effects [41,47,72,85]. This pattern indicates that extraction duration in maceration functions primarily as a compositional filter rather than as a simple driver of yield. Early extraction stages tend to enrich compounds that are both more soluble and more rapidly diffusible, whereas prolonged soaking permits gradual release of structurally bound or less accessible metabolites. At the same time, extended solvent exposure may alter the stability of sensitive compounds, further modifying the chemical profile of the extract. Consequently, differences in extraction time can produce biologically distinct macerates even when the same plant material and solvent system are used. In this context, variability in anticancer activity reflects not only how much material is extracted, but also how duration changes the relative balance between active, less active, and potentially interfering with constituents. A similar pattern is observed in studies using repeated maceration cycles. Re-extraction can improve recovery of metabolites that are not readily released during a single extraction step, thereby increasing extraction completeness [40]. However, this also increases chemical complexity, and the biological consequence is not uniform. In some instances, recovery of additional fractions has been associated with enhanced activity, whereas in others the broader composition appears to dilute the contribution of key bioactive constituents [39]. These findings further highlights that the impact of extraction duration is context-dependent rather than cumulatively advantageous. What matters is not prolonged extraction itself, but whether the additional recovery improves selective enrichment of pharmacologically relevant metabolites. Overall, the reviewed studies show that the influence of extraction duration in maceration is determined by how time alters extract composition and selectivity. Moderate durations often provide the most effective balance between recovery and selectivity, whereas prolonged extraction tends to increase yield at the risk of reducing compositional relevance. Accordingly, extraction duration in maceration should be interpreted as a selective and method specific parameter, not as a variable that can be extended indefinitely to improve bioactivity.

**Table 2:** Composition, bioactivity and anticancer potential of macerated phytochemicals.

Plant	Part (s) Used	Solvent	Analytical Method	Major Identified Compounds	Mechanisms	Target Organ/Cell Line and Assay	IC_50_ (µg/mL)	References
*Cannabis sativa* L.	Stem, branches, and trichomes	Ethanol	GC-MS, HPLC	Terpenoids: Monoterpene (nerol), sesquiterpene (α-Bisabol, guaiol), cannabinoids (cannabidiol acid (CBDA), cannabidiol (CBD)	Apoptosis induction, ROS generation and cell cycle arrest (G1)	Pancreas (AsPC-1)	AsPC-1 (80)	[74]
*Lavatera cashmeriana*	Leaf	Ethanol	GC-MS	Terpenoid (phytol), terpenoid derivative (2,6,10-trimethyl,14-ethylene-14-pen- tadecane), fatty acid (1-Eicosanol)	Cytotoxicity	Lung (A549), MTT assay	A549 (70.1)	[19]
*Eclipta alba*	Whole plant	Methanol	Spectrophotometry	Phenolics, flavonoids	Antiproliferation, apoptosis	Colorectal (HCT-116), prostate (PC-3), breast (MCF-7), and renal (RCC-45)	HCT-116 (179), MACF-7 (400), RCC-45 (498), and PC-3 (470)	[39]
*Teucrium polium* L.	Aerial	Methanol	UHPLC-PDA	Isoprenoid, fatty acids, amino fatty acids, amino alcohols, glycolipids, amino sugars, phenol, alkaloids, flavanol, small peptides	Antiproliferation	Breast (Walker 256/B), prostate (MatLyLu), MTT assay	Walker 256/B (20.1) and MatLyLu (25.8)	[40]
*Imperata cylindrica*	Root	Methanol	UHPLC-HRMS	Phenolics acid (caffeic acid), phenylpropanoid derivative (coniferyl acetate), alkaloids (ricinine), phenolic (epicatechin, curcumin, and myricetin)	Antiproliferation, Apoptosis and cell cycle arrest (G0/G1)	Cervix (HeLa and CaSki)	24, 48 and 72 h: HeLa (84.2, 75.1 and 68.0), CaSki (65.1, 55.5 and 50.7)	[79]
*Andrographis paniculata*	Leaf	Methanol	GC-MS	2(5H)-furanone, quinic acid, phytol	Cell-cycle arrest (G2/M), apoptosis (caspase-3), Bax upregulation, and Bcl-2 suppression, angiogenesis	Cervix (HeLa), breast (MCF7, BT549), kidney (HEK-293), and lung (A549)	HeLa (33.1), MCF7 (33.5), BT549 (43.8), HEK-293 (50.3), and A549 (36.2)	[80]
*Nepeta* species (*Nepeta trichocalyx*, *Nepeta cadmea*, *Nepeta crinata*, *Nepeta humilis*, *Nepeta italica*, *Nepeta leptentha*, *Nepeta meyeri*, *Nepeta phyllochlamys*, *Nepeta racemosa*, *Nepeta stricta var. curvidens*, *Nepeta stricta* *var. stricta*, *Nepeta teucriifolia*, *Nepeta transcaucasica*)	Aerial	Methanol	HPLC, spectrophotometry	Iridoid, triterpenoids, ursolic acid	Antiproliferation	Breast (MCF-7, and MDA-MB-231), MTT assay	*N. racemosa* (62.5) in MCF-7; *N. cadmea* (250), *N. crinata (250)*, *N. italica (250)*, and *N. phyllochlamys (250)* in MDA-MB-231; *N. argolica* (186.7), *N. stricta var. curvidens* (213.1), *N. leptentha* (200), *N. phyllochlamys* (79.9), *N. racemosa* (61.4), and *N. transcaucasica* (220.7) in MCF-7; *N. argolica* (166.1), *N. phyllochlamys* (90.8), *N. racemosa*, and *N. transcaucasica*, (94.3, and 178.1) in MDA-MB-231	[90]
*Nepeta nuda*	Leaf	Water	UHPLC-LTQ/UHPLC/qqqMS2	Phenolic acid (ferulic acid, rosmarinic acid, monoterpenoid (iridoid glycoside epideoxyloganic acid)	Autophagic cell death via ATG3/BECN1 upregulation and CASP8 activation	Breast (MDA-MB-231, MCF7), colon (HT29), liver (HepG2), MTT assay	MDA-MB-231 (481.6), MCF-7 (576.1), HT (504.2), HepG2 (541.4), Colon 26 (380.2)	[81]
*Drimia maritima*	Bulb	Water	GC-MS	Cardiac glycosides (proscillaridin A), phytosterol (stigmasterol, β-sitosterol, campesterol), elaidamide, terpenoid (limonene)	Apoptosis induction, ROS generation and TNF-α, IL-6, and CASP8 upregulation	Colon (COLO-205, Caco-2)	Caco-2 (0.9), COLO-205 (2.3)	[82]
*Citrus hystrix*	Leaf	Hexane	GC-MS	Terpenoids (citronellal, citronellol, oxygenated monoterpenes, hydrocarbon monoterpenes, oxygenated sesquiterpenes)	Apoptosis induction (Caspace-3), Bcl-2 downregulation, antiproliferation	Breast (MDA-MB-231)	MDA-MB-231 (317.6)	[76]
*Boascia coriacea*	Stem	Ethyl acetate	GC-MS	Triterpenoid (lup-20(29)-en-3-one and lupeol)	AR/Bcl-2 suppression with p53 upregulation, apoptosis (caspace-3)	Prostrate (DU-145)	DU-145 (32.2)	[77]
*Dodonaea viscosa Jacq*	Leaf	Hydro-ethanol	UHPLC-ESI-MS/MS	Flavonoids (naringenin, 4′,5,7-trihydroxy-3,6-dimethoxyflavone), terpenoid (Kauralexin B3)	Intrinsic apoptosis (caspase-3/p53) in SW620 and non-mitochondrial apoptosis in SW480	Colon (SW480, SW620)	SW480 24 h (129.8) 48 h, (87.7) and SW620 24 h (37.0) and 48 h (28.2)	[91]
*Bouea macrophylla*	Leaf	Hydro-ethanol	GC-MS, spectrophotometry	Polyphenol, flavonoids, caryophyllene, phytol, trans-geranylgeraniol	Apoptosis induction	Cervix (HeLa, HCT116), MTT assay	HeLa (24) and HCT-116 (28)	[42]
*Arctiumlappa* L., *Artemisia fragrans Willd. Chrysanthemum morifolium*, *Cichoriumintybus* L. *Cuscutaepithymum* L., *Equisetum arvensis* L. *Myrtuscommunis* L., *Urticadioica* L. *ArtemisiaA annua*	Aerial and root	Hydro-ethanol	Spectrophotometry	Phenolics, flavonoids	Apoptosis induction via TLR4–AKT–ERK–NF-κB pathway inhibition	Colon HT-29, KYSE, and AGS, MTT assay	10–10,000	[41]
*Smilax Ovalifolia*, *Desmodium triflorum*, *Chenopodium album*, *Sesbania grandiflora*, *Etlingera linguiformis*	Leaf	Hydro-ethanol	Spectrophotometry	Flavonoids, phenolics	Cytoprotection and ROS reduction	Breast (MCF-7, MDA-MB-231, MDA-MB-435S), liver (HepG2), MTT assay	MCF-7 (50.8), MDA-MB-231 (67.5), MDA-MB-4358 (122.1, 281.2, and 440.3)	[11]
*Vaccinium myrtillus* L.	Aerial (stem, leaf and fruits)	Hydro-ethanol	LC-Q-Orbitrap HRMS	Hydroxy-benzoic acids and derivatives (1 compound) hydroxycinnamic acids and derivatives (26 compounds), flavan-3-ols (6 compounds)	Cytotoxicity	Colon (HT29), breast (MCF-7), and cervix (HeLa), MTT assay	Not reported	[75]
*Musa paradisiaca*	Stem	Hydro-ethanol	GC-MS	Fatty acids (oleic acid, hexadecenoic acid, heptadecanoic acid, pentadecanoic acid)	Antiproliferation, apoptosis (caspase-3)	Oral (Hoscc), MTT assay	Hoscc (15.0)	[47]
*Becium grandiflorum*	Aerial	Hydro-ethanol	GC-MS, UPLC-PDA	Diterpene, (18-epoxy-pimara-8(14),15-diene, kauren-19-oic acid, isopimarol), phytosterol (stigmastadienol)	Apoptosis induction, cell-cycle arrest (G2/M), Bax–Bcl-2 pathway downregulation	Breast (MCF-7 and MDA-MB-231), colon (HCT-116), liver (HepG2), MTT assay	MCF-7 (43.5), HCT-116 (40.5), and HepG2 (19.9)	[2]
*Annona muricata* L.	Leaf	Hydro-ethanol	TLC	Flavonoids, antogenins	Bcl-2 suppression, apoptosis induction (caspase-9 and caspase-3)	Breast (MCF-7), lung (A549), cervix (HeLa),	MCF-7 (5.3), A549 (2.9), HeLa (3.1)	[87]
*Leontodon hispidulus Boiss.*	Aerial	Hydro-ethanol	HPLC, spectrophotometry	Flavonoids (catechin quercetin, rutin, kaempferol), phenolic acid (chlorogenic acid, ρ-coumaric acid, cinnamic acid, vanillic acid)	Cytotoxicity	Prostrate (PC3), cervix (HeLa)	PC-3 (16.5) and HeLa (23)	[54]
*Rhoicissus tridentata*	Root	Methanol, water	Spectrophotometry	Phenolics, flavonoids	Antiproliferation	Prostrate (LNCaP and DU 145), MTT assay	Methanol (377.9), water (359.4)	[92]
*Psoralea bituminosa*	Aerial	Methanol, water	LC-MS, spectrophotometry	Phenolic acids (4-hydroxybenzoic acid, caffeic acid, vanillin, ferulic acid, salicylic acid) flavonoids (daidzein, iso-orientin, vitexin, saponarin	Modulate OFOXPHOS, NRF2, TP53, PI3K, and MYC signaling pathways	Lung (A549), breast (MCF-7 and T47D, MDA-MB-231), Prostrate (PC-3 and DU145), colon (HCT-116), MTT assay	Methanol (27.7–53.9)	[83]
*Thymus daenensis* and *Stachys pilifera Benth*	Aerial	Hydro-methanol	GC-MS	Terpenoid (hexadeca-2,6,10,14-tetraen-1-ol, 3,7,11,16-Tetramethyl), fatty acid (linolenic acid)	Apoptosis induction (caspase-3)	Liver (HepG2), MTT assay	*T. daenensis*: 24 h (203.6), 48 h (210.2), 72 h (223.7), *S. pilifera* 24 h (128.50), 48 h (109.7) and 72 h (107.1)	[78]
*Prismatomeris glabra*	Leaf	Hydro-ethanol, methanol, and water	LC–MS QTOF	Triterpenoids (quillaic acid, tomentosic acid, ganoderic acid), flavonoids (epigallocatechin5,3′,5′-trimethylether3-o-gallate, silymarin, biochanin A)	Antiproliferation, apoptosis and cytotoxicity	Breast (MCF-7), MTT assay	Water 24 h (159.2), 48 h (110.9), and 72 h (158) ethanol 24 h (467.8), 48 h (251.5), 72 h (214.9), and methanol 24 h (318.3), 48 h (206.0), and 72 h (184.7)	[9]
*Vernonia amygdalina*	Leaf	Hexane and ethyl acetate	LC-MS/MS	Alkaloids (deacetyl vindoline), phenolics (licochalcone b), and steroids	Apoptosis induction (PTEN and XBP1 pathway)	Cervix (Hela), kidney (Vero) MTT assay	HeLa (0.7) and Vero (4.0)	[48]
*Arnebia nobilis*	Root	Ethanol, hexane	Spectrophotometry, GC-MS	Flavonoids, phenolics (deoxy-shikonin, ethyl cholate and 2-Methyl-6-(4-methylphenyl) hept-2-en-4-one), saponins, terpenoids	Apoptosis induction, angiogenesis	Liver (HepG2), MTT assay	Hexane (22.8) and ethanol (46.7)	[84]
*Hypericum perforatum*	Leaves and flowers	Dimethyl sulfoxide (DMSO), olive oil	Spectrophotometry, LC-MS/MS	Phenolic acids (vanillic acid, caffeic acid, chlorogenic acid, hydroxybenzoic acid), flavonoids (apigenin7-glucoside, quercetin)	Cytotoxicity	Prostrate (PC-3), MTT assay	PC-3 (0.21)	[88]
*Arthrocnemum macrostachyum* and *Tamarix nilotica*	Leaves and stem	Water	UHPLC-Q-ToF-MS, spectrophotometry	Terpene lactones (curcuma nose A; cinncassiol A), phenylacetic acids (homo-veratric acid), cyclohexanols (cis-4-hydroxycyclohexylacetic acid)	Cytotoxicity	Breast (MDA-MB231), colon (HCT-116), lung (A549), MTT assay	*T. nilotica* (88.6) and *A. macrostachyum* (95.0)	[18]
*Boerhavia diffusa* L.	Aerial	Dichloromethane, ethyl acetate, methanol, water	UHPLC-HRMS, and spectrophotometry	Phenolic acids (15 hydroxy-benzoic, hydroxycinnamic, phenolic acid), flavonoids, rotenoid, fatty acids	Cytoxicity	Breast (MDA-MB-231), MTT assay	Methanol: 24 h (582.9) and 48 h (304.7)	[85]
*Stellaria media*	Aerial	Ethanol, methanol, water, ethanol-water, methanol-water	Spectrophotometry	Flavonoids, phenolics, alkaloids, saponins, tannins	Antiproliferation, cell death and cytotoxicity	Breast (MCF-7), MTT assay	24 h (250), 48 h (180) and 72 h (150)	[86]
*Echinops shakrokii*	Aerial	Hydro-methanol, water, Ethyl acetate	LC-MS-QTOF	Flavonoids (catechin), stilbenes (resveratrol), coumarins (scopoletin), alkaloids (huperzine A), terpenoids (zerumbone) glycosides (leiocarposide), and organic acids (quinic acid)	Apoptosis induction (Caspase-3) immunostimulatory effects (Lymphocyte proliferation, macrophage phagocytic and pinocytic activities), antiproliferation	Breast (MCF-7, MDA-MB-231, EMT6/P, T47-D), kidney (VERO), colon (Caco-2), cervix (HeLa)	Ethyl acetate: MCF-7 (560) and hydro-methanol: T47D (500)	[89]

##### Impact of Maceration Conditions on Phytochemical Recovery

Comparison of maceration protocols, including static, cold, and agitated conditions, shows that phytochemical recovery is shaped not only by solvent choice and extraction time, but also by the physical conditions under which mass transfer occurs. Across the reviewed literature, agitated maceration is the most frequently applied approach, whereas purely static maceration is less commonly used and cold maceration is generally selected when preservation of thermolabile metabolites is prioritized [74]. This distribution reflects a clear methodological tradeoff between extraction rate, metabolite stability, and procedural simplicity. The predominance of agitated maceration is largely explained by its effect on diffusion dynamics. Mechanical shaking or stirring reduces the boundary layer surrounding plant particles and improves solvent penetration into plant tissues, thereby accelerating metabolite release into the solvent phase [39,86,89]. By contrast, static maceration relies entirely on passive diffusion and therefore proceeds more slowly, often requiring longer extraction periods to achieve comparable recovery [74]. However, slower extraction under static conditions does not necessarily imply inferior bioactivity. Rather, it indicates that similar phytochemical outcomes may be reached through different kinetic routes, depending on how extraction time, temperature, and solvent volume are balanced.

Static maceration also shows that extraction efficiency is influenced by more than agitation alone. Solvent to solid ratio, solvent type, and extraction temperature interact with mixing conditions to determine concentration gradients and metabolite mobility [74]. In this context, static systems may operate with lower solvent volumes, whereas agitated systems often use higher solvent volumes to sustain efficient diffusion. These differences affect extraction performance, but they do not establish a simple hierarchy of superiority. Instead, they show that maceration conditions alter the balance between solvent efficiency, extraction speed, and phytochemical recovery. Cold maceration provides a distinct but related trade off. Lower temperatures help preserve temperature sensitive compounds, but they also reduce diffusion rates and therefore require longer extraction times to achieve sufficient recovery [77]. This makes cold maceration slower than agitated extraction, yet potentially more suitable for compounds vulnerable to thermal degradation. Accordingly, the advantage of cold maceration lies not in faster recovery, but in preserving compositional integrity during prolonged extraction. Together, the reviewed studies indicate that static, cold, and agitated maceration should be understood as different kinetic and stability strategies rather than as simple procedural variants. Agitation primarily enhances diffusion and shortens extraction time, static maceration emphasizes operational simplicity and lower solvent demand, and cold maceration prioritizes preservation of thermolabile constituents while compensating through extended extraction. These differences are important as they shape not only extraction efficiency, but also the final chemical composition and bioactivity of macerates.

##### Fractionation Approaches in Maceration-Derived Extracts

A recurring limitation in the maceration literature is that many macerates are evaluated directly in cytotoxicity assays without subsequent fractionation or compound level characterization [19,43,74,80]. Although this approach is useful for rapid screening of anticancer potential, it limits mechanistic interpretation as the observed bioactivity remains attributable to crude extract systems rather than to clearly defined bioactive constituents. This lack of post extraction resolution also reduces comparability across studies. Differences in reported potency among macerates may reflect variation in crude extract complexity rather than true differences in intrinsic activity, particularly when extracts differ in solvent system, plant matrix, or extraction duration [19,40,74,79,81]. As a result, biological effects such as antiproliferative or apoptosis inducing activity can be observed without clear identification of the compounds primarily responsible. Incorporating targeted fractionation and compound identification after maceration would improve mechanistic clarity and strengthen cross-study comparison, while avoiding the need to treat crude extract activity as direct evidence of individual metabolite efficacy.

##### Variability in IC_50_ in Macerates

Variability in IC_50_ values reported for maceration-derived extracts should be interpreted in relation to the compositional heterogeneity of macerates rather than as a direct measure of extraction efficiency alone [2,9,11,18,19,39,40,41,47,48,54,73,74,75,76,77,78,79,80,81,82,83,84,85,86,87,88,89,90,91,92]. As discussed in the broader IC_50_ section, cytotoxic potency is influenced by biological model and assay conditions. However, within maceration studies, this variability is further amplified by the diffusion dependent and relatively low selectivity nature of the method. Since maceration often yields crude extracts containing broad and variably enriched mixtures of metabolites, reported IC_50_ values reflect not only intrinsic bioactivity, but also how solvent choice, plant matrix accessibility, extraction duration, and repeated extraction cycles shape the final chemical composition of the extract. This is particularly important as maceration does not actively intensify extraction through heat or pressure, and therefore recovery depends strongly on passive metabolite release from the plant matrix. Under these conditions, small differences in extraction time, solvent penetration, plant part, or tissue structure can substantially alter the relative abundance of active, less active, or potentially interfering constituents [2,9,11,18,19,39,40,41,47,48,54,73,74,75,76,77,78,79,80,81,82,83,84,85,86,87,88,89,90,91,92]. As a result, two macerates prepared using broadly similar conditions may still produce markedly different IC_50_ values if their chemical profiles differ in the extent to which cytotoxic metabolites are enriched or diluted. The interpretation of IC_50_ in maceration studies is also constrained by the frequent evaluation of macerates as crude extracts rather than as fractionated or chemically resolved samples. In such cases, the measured IC_50_ reflects the collective behavior of multicomponent phytochemical mixtures rather than the potency of clearly defined individual compounds. Within maceration studies, therefore, IC_50_ variability should be understood as a consequence of diffusion-mediated compositional heterogeneity superimposed on the usual biological and methodological sources of variation. This is why meaningful comparison of maceration-derived IC_50_ values requires attention not only to assay design, but also to the extraction conditions that shape the chemical composition of the crude macerate. 

#### 3.2.3. Percolation

Percolation is also well-established solvent-based extraction technique recognized for its continuous and efficient recovery of BACs from plant matrices [93,94,95]. Compared to maceration, its dynamic nature maintains a concentration gradient through continuous solvent renewal, thereby enhancing mass transfer and facilitating more complete extraction of target constituents [93,94,95]. The process typically involves pre-wetting (moistening), a short maceration phase, and subsequent solvent percolation through the plant material, allowing continuous collection of the extract. Its operation under ambient temperature conditions further makes it suitable for preserving thermolabile compounds such as polyphenols and alkaloids [95,96]. Despite these advantages, percolation was notably underrepresented in studies published between 2020 and 2025 investigating the anticancer potential of plant extracts. This contrasts with its established use in pharmaceutical extraction and highlights a disconnect between methodological capability and current research practice. Several factors may explain this limited adoption. Percolation typically requires larger solvent volumes, longer processing times, and careful control of particle size and flow dynamics to prevent channeling effects, which can compromise extraction efficiency [93,94]. In contrast, maceration offers simplicity and accessibility, while modern assisted techniques (e.g., ultrasound- or microwave-assisted extraction) provide faster extraction with reduced solvent consumption [93,94]. As a result, researchers may preferentially select methods that are less resource-intensive and more compatible with high-throughput experimental workflows. Additionally, the lack of standardized protocols and limited comparative studies evaluating percolation against other extraction methods in anticancer research may further contribute to its underutilization. This absence of method-specific data makes it difficult to assess its relative effectiveness in recovering BACs with anticancer activity. Nevertheless, the theoretical advantages of percolation suggest that it may still hold untapped potential for the recovery of structurally sensitive or low-abundance BACs. Future research should therefore consider systematic comparisons of percolation with both conventional and emerging extraction techniques to better define its role in phytochemical-based anticancer studies.

#### 3.2.4. Decoction

Decoction is a heat intensive aqueous extraction method in which sustained boiling promotes both the release and transformation of plant metabolites (Figure 4). Unlike infusion, which primarily facilitates diffusion of readily soluble compounds under milder conditions, decoction exposes plant material to prolonged thermal stress that can substantially alter extract composition [94,96]. Boiling disrupts plant tissues, improves solvent penetration, and enhances recovery of intracellular constituents, especially from structurally dense matrices. At the same time, it can degrade heat sensitive compounds, eliminate volatile constituents, and convert complex molecules into smaller or more stable derivatives [96]. Decoction should therefore be interpreted not simply as an aqueous recovery method, but as a reactive extraction environment in which extraction and chemical transformation occur simultaneously. This transformation driven character is central to understanding variability across studies [71,94]. As water is consistently used, differences in decoction-derived bioactivity are not primarily driven by solvent identity, but by how plant chemistry responds to sustained heating. Plant part remains important, yet its influence is expressed differently under boiling conditions. Dense tissues such as bark and roots become more accessible during decoction than under milder extraction methods, allowing release of metabolites that may otherwise remain embedded within the matrix [89,91]. Developmental stage adds a further layer of variability, since the concentration of precursors and thermally responsive metabolites changes during plant growth. As a result, decoction can recover a broader range of compounds, but not necessarily in their native form [52]. Heat induced processes such as hydrolysis, oxidation, and rearrangement can generate smaller and often more polar derivatives from more complex parent compounds. In some cases, this may enhance activity by producing more bioavailable or more reactive forms, whereas in others it may reduce activity through degradation of key constituents. The balance between these opposing effects is highly sensitive to boiling conditions, extraction duration, and plant matrix composition. This helps explain why cross-study comparison of decoction-derived extracts is inherently difficult, particularly when analytical depth differs and crude extracts are interpreted without distinguishing native metabolites from heat generated derivatives.

As previously discussed, IC_50_ variability reflects broader chemical, biological, and methodological factors. Reported IC_50_ values for decoction extracts span a wide range, and this should not be interpreted only as experimental inconsistency. Rather, it reflects that boiling can simultaneously reduce the concentration of potent native compounds and generate new derivatives with greater, lower, or simply different bioactivity. Accordingly, the effective concentration of bioactive molecules in a decoction may vary substantially even when total extract concentration appears similar. The predominance of polar constituents in decoction extracts adds another layer to this variability. Many water-soluble compounds have limited membrane permeability, which can reduce intracellular accumulation and lower apparent cytotoxic potency [52]. The measured biological response therefore reflects a balance between degradation, conversion, compound polarity, and cellular uptake rather than simple extract concentration alone [97,98]. When considered alongside other water- and heat-based extraction methods, the distinctive character of decoction becomes more apparent. In contrast to hydro-distillation, which primarily isolates volatile and relatively lipophilic constituents, decoction enriches nonvolatile, water-soluble metabolites. This divergence in extraction selectivity leads to fundamentally different chemical profiles and, consequently, distinct biological responses. Decoction extracts are more commonly associated with phenolic-mediated mechanisms, including oxidative stress modulation, apoptosis induction, and cell cycle regulation, whereas hydro-distilled extracts tend to exert membrane-targeted effects driven by volatile compounds. Overall, these differences underscore that extraction method shapes not only compound recovery but also the mechanistic pathways through which anticancer activity is expressed. This chemical selectivity also helps explain the relatively limited representation of decoction in anticancer phytochemical research. Prolonged boiling in water restricts the recovery of semi-polar and lipophilic compounds often prioritized in anticancer screening, while simultaneously increasing the risk of thermal degradation of heat-sensitive metabolites. Overall, decoction should therefore be understood not as a passive extraction process, but as a transformative aqueous system in which boiling drives both metabolite recovery and chemical reconfiguration. Future work should therefore focus on distinguishing native from heat-generated metabolites and on directly linking thermal transformation profiles to bioactivity.

#### 3.2.5. Infusion

Infusion, like decoction, is an aqueous extraction method widely used in traditional medicine for preparing herbal teas and medicinal beverages [71,90,94,99]. However, unlike decoction, infusion involves shorter exposure to boiling or near boiling water, usually for 15 to 30 min, and therefore represents a milder extraction process (Figure 5). This difference in thermal intensity directly influences both the phytochemical profile and the bioactivity of the resulting extracts. Since water is the sole solvent, infusion primarily recovers hydrophilic and moderately polar metabolites, including phenolic acids and flavonoid glycosides, while recovering relatively little of the lipophilic fraction of the plant metabolome. As a result, variation in bioactivity is driven less by solvent differences than by the selective nature of aqueous extraction and the characteristics of the plant material itself. Under infusion conditions, plant related factors strongly influence metabolite recovery. Soft tissues such as leaves and flowers generally allow more rapid diffusion of soluble compounds, whereas denser tissues such as roots and bark may restrict release under the shorter and less intensive conditions of infusion. Consequently, even when similar preparation protocols are used, extracts can differ substantially depending on plant part, tissue structure, and the distribution of soluble metabolites within the matrix. Thermal exposure introduces an additional but more moderate layer of variability. Although the heat used during infusion enhances release of intracellular constituents, it can also alter or partially degrade thermolabile compounds [93]. These effects are less extensive than in decoction, but they still contribute to differences in the final extract profile across studies. The bioactivity of infusion extracts is therefore best understood as the result of selective recovery of water-soluble metabolites rather than exhaustive extraction of the full phytochemical spectrum. In many cases, activity reflects the combined action of multiple low to moderate activity constituents rather than strong enrichment of a few highly potent compounds [90]. This helps explain why infusion extracts often show modest and variable cytotoxic effects in vitro. The issue is not simply that infusion is weaker, but that its aqueous selectivity tends to reduce recovery of highly cytotoxic semi-polar and lipophilic constituents that are often emphasized in anticancer screening.

Within infusion studies, IC_50_ variability is further shaped by the limited intracellular delivery of polar constituents. Reported IC_50_ values are generally high and variable [85,93], and this should not be interpreted only as evidence of low intrinsic activity. Rather, infusion extracts frequently contain lower concentrations of highly potent lipophilic compounds and are dominated by water soluble metabolites that may have more limited membrane permeability. As a result, the effective intracellular dose of bioactive constituents may remain low even when similar total extract concentrations are applied. In this context, measured IC_50_ values reflect not only extract chemistry but also compound uptake and the interaction between a selectively recovered polar mixture and a specific biological model. Comparison with hydro-distillation further clarifies the distinctive profile of infusion extracts. Whereas infusion recovers mainly non-volatile, water-soluble metabolites, hydro-distillation isolates volatile and relatively lipophilic constituents such as essential oils. These chemical differences lead to distinct biological patterns. Hydro-distilled extracts are often associated with membrane related and oxidative mechanisms driven by volatile constituents whereas infusion extracts more commonly reflect the activity of polar metabolites involved in oxidative stress modulation, apoptosis, and related signaling pathways. This comparison shows that extraction method shapes not only which compounds are recovered, but also which types of anticancer responses are most likely to be observed. Infusion remains less represented in anticancer phytochemical research largely owing to its mild aqueous conditions recover a narrower range of metabolites than organic solvent based or intensified extraction methods. Studies designed to maximize phytochemical diversity or isolate highly potent semi polar and lipophilic compounds therefore tend to favor other techniques. This preference also helps explain why aqueous methods such as infusion and, to some extent, decoction is less frequently used in anticancer screening, despite their relevance to traditional medicine. However, infusion should not be regarded simply as an inferior extraction method. Its selectivity may better reflect physiologically relevant exposure to plant-derived compounds as they are commonly consumed, and it may favor constituents that are safer, more bio accessible, and more representative of real-world use. Infusion is best seen as a mild and selective aqueous extraction method that favors recovery of water-soluble metabolites under relatively limited thermal exposure. Its variability in anticancer activity arises mainly from plant matrix dependent diffusion, selective enrichment of polar compounds, and the often-low intracellular delivery of the recovered constituents. Future studies should better define the relationship between infusion chemistry, cellular uptake, and biological response, particularly in models that reflect physiologically relevant exposure.

#### 3.2.6. Soxhlet Extraction

Soxhlet extraction is a dynamic solvent extraction method in which condensed solvent repeatedly percolates through plant material under continuous reflux (Figure 6). Compared with diffusion based techniques, this repeated solvent renewal improves mass transfer and supports more exhaustive recovery of soluble metabolites [20,22,34,35,37,38,49,89,100,101]. However, since extraction occurs under sustained heating, Soxhlet does not recover all metabolites equally. Compounds that remain stable during repeated reflux are more likely to be enriched, whereas heat sensitive constituents may be degraded or underrepresented [102]. Accordingly, the phytochemical profile and anticancer activity of Soxhlet derived extracts reflect not only plant chemistry, but also the combined effects of solvent choice and thermal exposure.

##### Solvent Polarity and Phytochemical Selectivity in Soxhlet Extraction

Under Soxhlet conditions, solvent polarity operates within a reflux environment that favors repeated solubilization of compounds able to withstand sustained heating. As a result, Soxhlet extraction does not recover metabolites based on solvent compatibility alone but preferentially enriches constituents that are both soluble and thermally stable.

Across the reviewed studies, ethanolic, methanolic, ethyl acetate, and sequential Soxhlet systems recurrently yielded metabolite classes such as sterols, fatty acids, terpenoids, alkaloids, and phenolic acids [20,22,34,35,37,38,49,89,100,101]. The recurrence of these groups across taxonomically distinct plants suggests that Soxhlet extraction exerts a methodological filtering effect that enriches relatively thermostable and moderately lipophilic constituents. This selectivity is important for interpreting bioactivity. Activity depends on whether the extraction conditions enrich the metabolites most relevant to anticancer effects. Therefore, differences in the bioactivity of Soxhlet derived extracts should be interpreted in relation to solvent dependent recovery under reflux conditions rather than solvent identity alone.

##### Influence of Extraction Cycles on Phytochemical Recovery

In Soxhlet extraction, the number of solvent cycles is a major determinant of extraction completeness as each cycle exposes the plant matrix to newly condensed solvent and promotes further dissolution of less accessible compounds [49]. Increasing cycle numbers therefore broadens phytochemical recovery, but it does not necessarily improve extract selectivity. As additional cycles recover a wider range of metabolites, they may also increase the proportion of constituents that contribute little to anticancer activity, thereby weakening the relative enrichment of the most active compounds. Cycle dependent changes in composition are further shaped by sustained reflux. Repeated thermal exposure favors thermostable metabolites, while more sensitive constituents may be altered or lost during extraction. Thus, the effect of cycle number is not simply cumulative, but compositional. Where downstream fractionation has been applied, stronger activity has often been localized to specific fractions rather than to the crude Soxhlet extract [49].This indicates that broader recovery through extended cycling can mask selectively active constituents within chemically complex mixtures. Accordingly, Soxhlet cycle number should be interpreted as a determinant of extract composition rather than as a direct predictor of biological potency.

##### Variability in IC_50_ Across Soxhlet-Derived Extracts

Across Soxhlet extraction studies, reported IC_50_ values for derived extracts ranged from 12.4 to 800 µg/mL (Table 3), indicating substantial variation in cytotoxic potency across studies. This variation is best understood in relation to how solvent system, plant matrix, thermal exposure, and cycle number influence the relative abundance of thermostable bioactive constituents in the final extract. Since Soxhlet extraction favors metabolites compatible with both solvent and heat, small differences in tissue chemistry, cycle number, or reflux exposure can alter whether cytotoxic constituents are enriched, diluted, or thermally modified. In addition, many Soxhlet derived extracts are evaluated as crude mixtures rather than chemically resolved systems, so the measured IC_50_ reflects the collective behavior of multicomponent extracts rather than the potency of clearly defined individual metabolites. Therefore, cross-study comparison of Soxhlet derived potency should be made in the context of solvent system, reflux conditions, cycle number, plant matrix, and the level of downstream chemical resolution. Overall, Soxhlet extraction is best viewed as an exhaustive but thermally selective extraction method that enriches compounds compatible with both solvent environment and sustained reflux. Variability in bioactivity arises mainly from the combined effects of solvent dependent selectivity, cycle number, and thermal filtering of sensitive versus thermostable metabolites. Future studies should prioritize tighter control of reflux conditions and integrate compound-level characterization to distinguish true bioactive enrichment from heat driven compositional bias.

#### 3.2.7. Hydro- and Steam Distillation

Hydro- and steam distillation remain relatively underrepresented in phytochemical anticancer research, with only a limited number of studies in the reviewed literature [103] (Figure 7). This limited use reflects the distinct chemical focus of the method, which primarily isolates volatile and lipophilic constituents such as essential oils rather than the broader pool of non-volatile phytochemicals, including phenolics, flavonoids, and alkaloids, that are more commonly emphasized in anticancer screening [16,103]. Traditional distillation also involves prolonged heating and relatively high energy input, which can reduce recovery or promote partial loss of heat sensitive volatiles. As a result, hydro- and steam distillation occupy a different methodological and chemical space from solvent based extraction approaches [103]. Despite the limited evidence base, a consistent pattern emerges in which bioactivity appears to depend more on the composition of the essential oil fraction than on modest differences in extraction duration. Within the available studies, hydro-distilled fractions produced relatively comparable cytotoxic responses despite differences in processing time, suggesting that compound profile is more decisive than minor variation in distillation conditions. This pattern is important as it distinguishes hydro- and steam distillation from methods in which broader crude extract heterogeneity often dominates biological interpretation.

A notable feature of hydro-distilled extracts is their comparatively defined chemical composition. Essential oils are typically enriched in volatile lipophilic constituents, which reduces the dilution effect commonly seen in crude solvent extracts that contain many inactive or low activity compounds. This more restricted chemical profile may help explain why reported IC_50_ values are moderate and appear less variable than those often observed for chemically complex crude extracts. In this context, narrower IC_50_ variation should not be interpreted as evidence of greater general potency, but rather due of selective enrichment of a smaller class of active membrane metabolites. The mechanistic profile of these extracts is also relatively consistent. Reported cytotoxic effects are most associated with reactive oxygen species generation and apoptosis induction [6,17], which is plausible given the physicochemical properties of major volatile constituents such as thymol, carvacrol, and limonene. Since these compounds are highly lipophilic, they can penetrate cellular membranes efficiently, accumulate within cells, and promote oxidative stress mediated disruption of mitochondrial function [6,17,103]. Compared with polar extracts, where limited membrane permeability may reduce effective intracellular exposure, hydro-distilled fractions may achieve more direct interaction with intracellular targets. This provides a mechanistic basis for the comparatively consistent cytotoxic responses observed across the limited number of studies. Within hydro- and steam distillation studies, however, variability appears to be shaped less by extraction duration and more by essential oil composition, membrane permeability, and experimental context. Since the extracted fractions are chemically narrower than many crude solvent extracts, the measured IC_50_ values are influenced less by co extracted inactive constituents and more by the abundance of specific volatile BACs. Even so, interpretation remains constrained by the small number of studies and by differences in mechanistic assays, model systems, and analytical depth, which limit direct comparison and prevent strong structure activity conclusions. These findings reinforce that extraction method plays a defining role in shaping both the chemical profile of plant extracts and the biological responses they produce. Overall, hydro- and steam distillation represent chemically focused extraction strategies that selectively enrich volatile, lipophilic constituents with relatively direct membrane related biological effects. Variability across studies appears to depend less on minor processing differences and more on essential oil composition, compound membrane-related experimental context. Future progress in this area will require broader phytochemical characterization of distilled fractions and more standardized biological assays to clarify structure activity relationships among volatile BACs.

**Table 3:** Composition, bioactivity and anticancer potential of Soxhlet-extracted phytochemicals.

Plant	Part (s) Used	Solvent	Analytical Method	Major Identified Compounds	Mechanisms	Target Organ/Cell Line and Assay	IC_50_ (µg/mL)	References
*Solanum trilobatum*	Leaf	Ethanol	GC-MS	Fatty acids (lauric acid, oleic acid, stearic acid), phytosterol (stigmasterol), terpenoid (phytol), tocopherol(α-tocopherol)	Cytotoxicity and cell inhibition	Cervix (HeLa and HCT116), MTT assay	HeLa: 83.14 and HCT116: 115.23	[100]
*Astragals spinosus*	Roots	Ethanol	GC-MS, spectrophotometry	Alkaloids, terpenoids, flavonoids, phenols, carboxylic acids, furans, and alkene derivatives	Cell inhibition	Breast (MCF-7), MTT assay	MC-7 (23.5)	[34]
*Andrographis paniculata*, *Centella asiatica*, *Psidium guajava*, and *Solanum trilobatum*	Leaf	Ethanol	GC-MS	Alkaloids, terpenoids, steroids, fatty acids and phenolics	Antiproliferative	Cervix (HCT116 and HeLa), MTT assay	*A. paniculata* (HeLa: 27.5 and HCT116: 30.2) and *S. trilobatum* (HeLa: 32.4 and HCT116: 35.5)	[22]
*Vernonia amygdalina*	Aerial	Ethanol	TLC	Flavonoids, alkaloids, tannins, saponins, glycosides	Apoptosis induction, cell-cycle arrest (G2/M), proliferation	Breast (MCF-7/HER-2), MTT assay	Ethanol: MCF-7 (130), and ethyl acetate: MCF-7 (66)	[20]
*Thymbra sintenisii*	Leaf	Ethanol	GC-MS, spectrophotometry	Terpenoid (lupeol acetate), fatty acid (stearic acid and linoleic acid)	Reduced cell viability and cytotoxicity	Breast (MCF-7)	MCF-7 (27.2)	[21]
*Avicennia alba*	Leaf	Methanol	GC-MS	Terpenoid (phytol), silicones (cyclohepta-siloxane), phenolics (2-Methoxy-4-vinylphenol) and fatty acid (hexadecanoic acid)	Reduced cell viability	Breast (MCF7) and cervix (HeLa), MTT assay	MCF7 (57.0) and HeLa (44.3)	[35]
*Asystasia gangetica*	Leaf	Petroleum ether and hydro-ethanol	LC-MS/MS, TLC, spectrophotometry	Phenolic acids (caffeic acid, cinnamic acid) and flavonoids (luteolin, catechin)	Apoptosis induction, cell-cycle arrest (G2M phase)	Colon (HCT-116), prostate (PC-3), cervix (HeLa), MTT assay	HCT-116 (24 h: 43.8, 48 h: 13.5), HeLa (24 h: 55.0), PC-3 (24 h 73.4)	[49]
*Strobilanthes ciliatus*	Leaf	Ethanol and acetone	GC-MS	Terpenoids (phytol, squalene), fatty acids (hexadecanoic and octadeconoic acid, eicosanoid acid)	Apoptosis and cell cycle arrest (G0/G1)	Colon (HT-29), MTT assay	Ethanol: HT-29 (33.6), acetone: HT-29 (105.2)	[36]
*Eryngium caucasicum*	Aerial	Hexane, DCM and methanol	GC-MS	Fatty acids (hexadecanoic acid), fatty acid esters (2-hydroxy-methyl ester), and terpenoid alcohols (trimethylbicyclooctane-2-ol)	Apoptosis and necrosis	Skin (B16F10)	24 and 48 h: Hexane: B16F10 (14.6, 11.0), DCM: B16F10 (31.2, 24.8) and MeOH: B16F10 (47.6, 36.4)	[101]
*Artemisia marschalliana*	Shoots	Hexane, DCM and methanol	GC-MS	Terpenoids: Monoterpenes (camphene, camphor), sesquiterpenes (copaene, germacrene D), diterpenoid (phytol, epimanoyl oxide), phenylpropanoids (eugenol, methyl cinnamate)	Apoptosis induction and antiproliferation	Breast (MCF-7, SW872), MTT assay	MCF-7: 24 h (17.9–800), 48 h (9.4–800), SW872 (24 h) (43.7–800) and 48 h (38.5–800)	[37]
*Eichhornia crassipes*	Leaf, roots, petioles	Ethanol, chloroform, acetone, methanol, and water	NMR, spectrophotometry	Alkaloids, flavonoids, steroids, terpenoids	Growth inhibition	Skin (SK-Mel-5), MTT assay	172.2	[38]
*Citrullus colocynthis*	Fruits	Hexane, dichloromethane, ethyl acetate, and butanol	HPLC-ESI-QTOF-MS/MS	Phenolic acids (ferulic acid, syringic acid, vanillic acid), cucurbitin derivative (cucurbita-5(10),6,23-triene-3β,25-diol), flavonoids (kaempferol 3-o-(6”-o-acetyl) glycoside, myricetin-3-o-glucoside)	EGFR inhibition	Pancreas (MIAPaCa-2) and skin (A-431), MTT assay	Ethyl acetate: MIAPaCa-2 (12.4) and A-431 (19.1)	[46]
*Clerodendrum infortunatum*	Aerial: Leaf	Water and hydro-ethanol	HPTLC	Phenolics (rutin, quercetin, gallic acid)	Apoptosis induction, S/G2–M arrest, ROS reduction, and BCL-2 signaling modulation	Cervix (HeLa)	HeLa: 242.3	[33]
*Echinops Shakrokii*	Aerial	Ethyl acetate	LC-MS-QTOF	Flavonoids (catechin), stilbenes (Resveratrol), coumarins (scopoletin), alkaloids (huperzine A), terpenoids (zerumbone) glycosides (leiocarposide), and organic acids (quinic acid)	Apoptosis induction (Caspase-3) immunostimulatory effects (Lymphocyte proliferation, macrophage phagocytic and pinocytic activities), antiproliferation	Breast (MCF-7, MDA-MB-231, EMT6/P, T47-D), kidney (VERO), colon (Caco-2), cervix (HeLa)	Ethyl acetate: MCF-7 (560) and hydro-methanol: T47D (500)	[89]

### 3.3. Integrative Framework of Anticancer Activity Across Extraction Methods

To improve cross-study synthesis, Table 4 groups reviewed studies by cancer model and integrates the dominant phytochemical classes, extraction approaches, mechanisms, and representative bioactivity patterns associated with each group.

## 4. Comparative Evaluation of Solvent-Based Extraction Techniques

**Figure 8:**
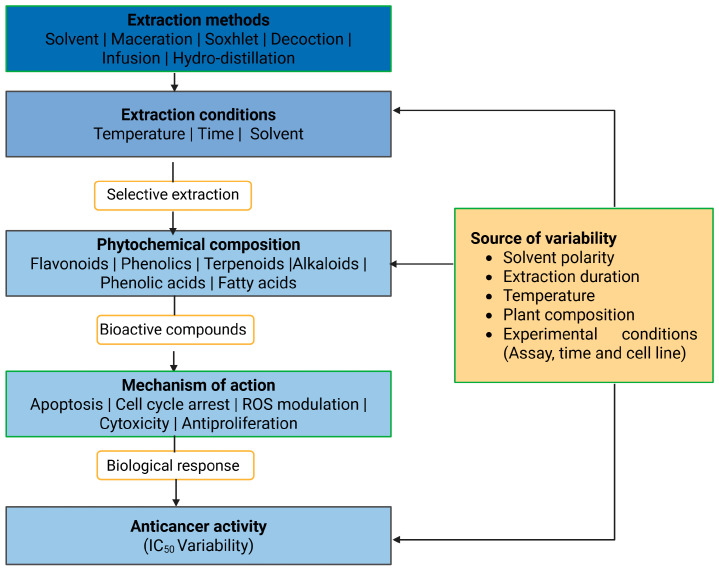
Extraction-driven variability in phytochemical composition and anticancer activity. Created with BioRender.

Comparative evaluation of the solvent-based extraction methods reviewed in this study indicates that no single technique consistently produces superior anticancer activity across medicinal plants. Rather, each method shapes phytochemical recovery through a distinct combination of solvent environment, extraction intensity, thermal exposure, and mass transfer conditions (Figure 8). In this sense, solvent-based extraction techniques function primarily as upstream modulators of phytochemical availability and composition, whereas the bioactivity ultimately observed depends on the identity, abundance, stability, and interaction of the recovered BACs rather than on the extraction label alone.

As summarized in Table 5, several comparative patterns emerge across methods. Diffusion based techniques such as maceration generally preserve thermolabile constituents but often yield chemically broad and variably selective crude extracts, making bioactivity highly sensitive to solvent choice, extraction duration, and plant matrix accessibility. Heat intensive methods such as decoction and Soxhlet extraction improve recovery from dense or poorly accessible tissues, yet they also introduce method specific constraints through thermal degradation, compositional filtering, or heat induced transformation of metabolites. By contrast, hydro- and steam distillation recover chemically narrower fractions enriched in volatile lipophilic constituents, which may contribute to more consistent membrane-related biological effects while simultaneously restricting the range of recovered phytochemicals. These patterns indicate that cross method differences are expressed primarily through selectivity, stability, extract complexity, and chemical transformation rather than through a simple hierarchy of potency.

A key conclusion of this review is therefore that extraction efficiency and anticancer efficacy are not directly correlated. Methods that recover more constituents do not necessarily enrich the compounds most relevant to cytotoxic or antiproliferative activity, and broader phytochemical recovery may dilute the contribution of key bioactive constituents. Likewise, similar extraction protocols can yield substantially different bioactivity as plant species, plant part, tissue structure, developmental stage, and extraction conditions all shape the final chemical profile. This explains why wide variability in reported IC_50_ values is observed both within and across extraction methods, and why nominal methodological similarity does not ensure biological comparability. Another major comparative insight is that post extraction analytical depth often determines interpretive strength more strongly than extraction technique alone. Studies that incorporate fractionation and compound level characterization provide clearer mechanistic attribution and stronger structure activity interpretation than those evaluating crude extracts alone, regardless of the initial extraction method. Consequently, differences in reported potency may reflect not only how the extract was produced, but also whether activity was assessed in chemically complex crude mixtures or in selectively enriched fractions. This uneven level of analytical resolution remains a major source of asymmetry across literature. From a translational perspective, the most important question is not which solvent-based extraction method is universally best, but which method most appropriately aligns extraction conditions with the physicochemical properties, stability, and intended biological targets of the metabolites of interest. Future progress will therefore depend on developing standardized extraction analysis workflows that integrate reproducible processing conditions, defined chemical markers, and biologically relevant validation systems [24,25,26].

Greater use of three-dimensional culture models, in vivo studies, and compound linked bioactivity analysis will be essential for improving both mechanistic clarity and cross-study comparability [74]. Overall, the reviewed literature shows that solvent-based extraction methods should be evaluated not as competing routes to a single optimal extract, but as different strategies for sampling, preserving, modifying, or enriching particular regions of plant chemical space. Their comparative value lies in how effectively they align recovery conditions with the stability and biological relevance of target metabolites. Accordingly, more meaningful progress in phytochemical anticancer research will depend on phytochemical informed and biologically validated method selection rather than on the assumption that any one solvent-based extraction technique is inherently superior.

## 5. Challenges in Solvent-Based Extraction Techniques

A major challenge in evaluating solvent-based extraction techniques is that differences in operational conditions do not translate into simple or consistent differences in anticancer activity. Across the reviewed literature, extracts obtained by maceration, Soxhlet extraction, decoction, infusion, and hydro- or steam distillation showed wide variation in cytotoxic potency, with both highly active and weak extracts reported within the same method category. This indicates that extraction method alone is not a reliable predictor of bio efficacy and that direct comparison among techniques is complicated by several interacting sources of variability. One important source of this variability is method specific selectivity. Conventional extraction methods differ in solvent environment, extraction intensity, thermal exposure, and mass transfer dynamics, all of which influence which metabolites are recovered, preserved, transformed, or underrepresented. However, these differences do not generate a simple hierarchy of biological effectiveness. Similar classes of phytochemicals may be recovered across different methods when comparable extraction media are used, while the same method may yield markedly different bioactivity when applied to different plant matrices. A further challenge is the strong influence of plant related variability. Plant species, plant part, tissue structure, developmental stage, and post-harvest condition can all substantially alter phytochemical composition before extraction begins. Consequently, extracts prepared using similar protocols may show very different IC_50_ values and bioactivity simply because the starting material differs in the abundance and distribution of bioactive constituents. This makes it difficult to separate the effect of extraction methods from the effect of plant chemistry itself and remains a major obstacle to meaningful cross-study comparison.

Another persistent challenge is the lack of standardization. Even within the same extraction category, substantial variation exists in solvent composition, extraction duration, temperature, solvent to solid ratio, and downstream processing. Without validated and reproducible extraction analysis pipelines, it remains difficult to achieve batch to batch consistency or to compare results across laboratories. Standardization is therefore not only a methodological issue, but also a prerequisite for linking phytochemical composition to biological function in a reliable and translationally meaningful way. Finally, biological validation remains limited, although some studies have begun to incorporate more advanced models, the overall lack of integrative validation frameworks reduces the translational relevance of many reported findings. Overall, these challenges show that solvent-based extraction methods cannot be judged based on yield or reported IC_50_ values alone. Their evaluation requires consideration of method specific selectivity, plant source variability, analytical resolution, and biological validation depth. These limitations help explain why cross-study comparisons remain difficult and why a stronger comparative framework is needed to assess the real impact of extraction strategy on anticancer activity.

## 6. Conclusions and Future Perspectives

BACs derived from medicinal plants show considerable anticancer potential, but this review demonstrates that their observed activity is inseparable from the extraction strategies used to recover them. Extraction is not merely a preparative step; it is a decisive factor that determines which region of plant chemical space is sampled, which compounds are preserved or transformed, and which biological responses can ultimately be observed. Across the literature, variability in solvent environment, extraction intensity, thermal exposure, plant matrix accessibility, and analytical resolution collectively shapes phytochemical profiles and reported anticancer outcomes. A central overarching insight emerging from this review is that variability in reported anticancer activity particularly IC_50_ values is fundamentally driven by extraction-dependent differences in phytochemical composition, rather than biological response alone. Wide variation in IC_50_ values across studies reflects not only biological response, but also differences in plant material, extraction conditions, selective enrichment, compound stability, and the level of post-extraction chemical resolution. In many cases, extraction efficiency and anticancer efficacy are not directly aligned; methods that recover more material do not necessarily enrich the metabolites most responsible for cytotoxic or antiproliferative effects, and crude extract systems cannot be interpreted as equivalent to chemically resolved fractions. A more meaningful evaluation of phytochemicals anticancer potential therefore requires closer integration of extraction design, phytochemical characterization, and biological testing. A further key insight is that no single solvent-based extraction technique is universally superior. Instead, each method offers a distinct balance among selectivity, recovery, stability, and compositional modification. Diffusion-based methods tend to preserve thermolabile constituents but often yield chemically broad crude mixtures, whereas heat-intensive methods can improve recovery from dense tissues while also driving degradation, selective loss, or transformation of metabolites. Distillation-based methods recover narrower volatile fractions with more defined membrane-related effects but sample only a limited portion of the phytochemical spectrum. The biological efficacy reported across studies is therefore governed less by the name of the extraction method than by the qualitative and quantitative composition of the recovered metabolites and the degree to which those metabolites remain stable, bioaccessible, and mechanistically relevant. Addressing these challenges requires a shift toward a unified, phytochemical-informed framework that integrates extraction design, chemical characterization, and biological validation. Future progress in this field will depend on three interconnected priorities. First, extraction workflows must become more standardized and phytochemical-informed, supported by validated extraction-analysis pipelines, reproducible processing conditions, defined marker compounds, and quality control frameworks that link chemical composition to biological function. Second, biological interpretation must move beyond reliance on crude extract potency alone, with greater emphasis on fractionation, compound-level bioactivity mapping, and mechanistic validation to identify the constituents or combinations responsible for anticancer effects. Third, experimental validation must extend beyond solvent-based two-dimensional cell culture models toward more biologically relevant systems, including three-dimensional cultures, in vivo studies, and integrated assessments of bioavailability, metabolism, and toxicity. Within this framework, emerging technologies can accelerate progress when integrated into a coherent and hypothesis-driven strategy rather than treated as isolated innovations. Hybrid extraction approaches that combine solvent-based and assisted methods may enhance selectivity, recovery, and compound stability when guided by phytochemical objectives. Artificial intelligence, automation, and real-time process monitoring offer additional opportunities for predictive optimization of extraction conditions. Likewise, integrated databases linking plant species, extraction parameters, phytochemical profiles, and bioactivity could strengthen reproducibility and enable more meaningful cross-study comparisons. However, the value of these advances will depend on whether they are anchored in rigorous chemical validation and biologically relevant testing, rather than on extraction efficiency alone. Overall, the future of anticancer phytochemical research will depend not on identifying the single best extraction method, but on aligning extraction strategy with metabolite chemistry, mechanism of action, and translational intent. Such an approach will enable the field to move beyond exploratory screening toward reproducible, mechanism-driven, and clinically translatable applications of plant-derived BACs.

## Figures and Tables

**Table 4:** Integrative framework grouping reviewed studies by cancer model and summarizing associated phytochemical classes, extraction approaches, mechanisms of action, and reported bioactivity.

Cancer Type	Representative Cell Lines	Dominant Phytochemical Class	Common Extraction Methods	Mechanism of Action	Bioactivity (IC_50_)	References
Breast	MCF-7, Walker 256/B, MDA-MB-231, MDA-MB-435, HER-2, MDA-MB-468, MCF-10A, 4T1, SW872, BT549, BT-20 EMT6/P, T47-D	Phenolics, phenolic acids, flavonoids, terpenoids, saponins and alkaloids	Solvent, Maceration, Soxhlet, hydro-distillation and infusion	Apoptosis induction (Caspase 3), cytotoxicity, antiproliferation, cell cycle arrest (G2/M) and Bax-Bcl pathway upregulation/downregulation	Strong–weak	[2,3,4,9,10,11,18,20,21,22,31,34,35,37,39,40,43,44,53,59,60,61,62,64,66,68,70,71,75,76,81,83,85,86,87,89,90]
Lung	HepG2, MRC5-SV2, A549, H1573, H1437, NCI-H2126, WI-38	Flavonoids, terpenoids, phenolics and phenolic acids	Solvent, Maceration, infusion	Apoptosis induction, cytotoxicity, antiproliferation, ROS, Bcl-2 downregulation	Strong–moderate	[1,22,32,42,60,63,65,66,83]
Colon	Caco-2, HT-29, HCT-116, DLD-1, HCT-15, SW480, COLO-205, SW620,	Flavonoids, phenolics, terpenoids, phenolic acids, alkaloids, fatty acids	Solvent, Soxhlet, maceration, decoction	Apoptosis induction, CASP8 activation, cell cycle arrest, antiproliferation, cytotoxicity, IL-6/IL-8 expression	Strong–weak	[2,4,6,18,36,39,41,45,49,50,51,52,53,60,66,71,75,81,82,89,91]
Liver	NCI-H460, HepG2,	Flavonoids, phenolics, terpenoids	Solvent, Maceration, decoction	Apoptosis induction, angiogenesis	Strong–weak	[10,42,66,71,78,84]
Prostate	MatLyLu, DU-145, PC3, LNCaP	Phenolic acids, flavonoids, terpenoids	Maceration, Soxhlet, solvent	Cell cycle arrest, antiproliferation, apoptosis induction	Strong–moderate	[39,40,49,77,88,92,103]
Cervix	Hela, CaSKi, SiHa HPV-16, HCT-116, Hep-2	Phenolic acids, phenolics and flavonoids	Maceration, solvent, decoction	SMAC activation, apoptosis induction, antiproliferation, cytotoxicity	Strong–moderate	[1,6,12,20,22,33,44,49,66,69,71,79,80,87,89,100]
Pancreas	AsPC-1, MIA PaCa-2, PaTu 8902,	Phenolic acids, organic acids, terpenoids	Maceration, solvent, Soxhlet	Apoptosis induction, ROS, cell cycle arrest, STAT4/PIK3CD gene expression	Strong–weak	[5,46,74]
Kidney	HEK-293 VERO, T24	Phenolic acids, flavonoids	Maceration, Soxhlet, infusion	Apoptosis induction, antiproliferation, cell cycle arrest	Strong–moderate	[6,48,80,89]
Oral	Hoscc	Fatty acids	Maceration	Antiproliferation, apoptosis induction	Strong	[47]
Renal	RCC-45	Phenolics, flavonoids	Maceration	Apoptosis induction, antiproliferation	Weak	[39]
Skin	B16F10, SK-Mel-5, A-431, A2058	Flavonoids, terpenoids, fatty acids	Soxhlet, solvent	Necrosis, apoptosis, EGFR inhibition and antiproliferation	Strong–weak	[38,46,51,101]
Stomach	AGS	Ovatodiolide	Solvent, decoction	G2/M cell cycle arrest, Bax upregulation, Bcl downregulation	Strong	[67]
Brain	T98G	Flavonoids, terpenoid and phenolics	Solvent	Cytotoxicity	Strong	[61]
Bladder	RT-4	Flavonoids, terpenoid and phenol-ics	Solvent	Cytotoxicity	Strong	[61]
Larynx	Hep-2	Flavonoids, terpenoids and tannins	Solvent	Antiproliferation	Moderate	[66]

**Table 5:** Comparative analysis of solvent-based extraction methods and their influence on phytochemical composition and anticancer activity.

Extraction Method	Extraction Principle	Temperature	Extraction Duration	Dominant Phytochemicals	Selectivity	Extraction Efficiency	Thermal Degradation	Bioactivity (IC_50_)	Mechanism of Action	Variability	Key Limitation	References
Solvent	Differential solubility based on solvent polarity	Ambient	Moderate (24–48 h)	Phenolic acids, alkaloids, flavonoids, stilbenes, terpenoids and phenolics	Variably selective depending on solvents and require further separation and purification	Moderate-high; dependent on solvent	Low	Highly variable; dependent on solvent polarity and phytochemical composition	Apoptosis induction (Caspace-3), cell cycle arrest (G2/M), Bax upregulation and Bcl-2 downregulation, antiproliferation and cytotoxicity	Solvent polarity and composition	Co-extraction of multiple compounds requiring further purification	[1,3,4,5,6,10,12,21,31,32,43,44,45,50,51,52,53,59,60,61,62,63,64,65,66,67,68,69,70]
Maceration	Passive diffusion driven mass transfer with shaking over time until solid–liquid equilibrium is reached	Ambient	Long (h to wk)	Flavonoids, terpenoids, phenolic acids, fatty acids and alkaloids	Insufficiently selective requires further separation and purification	Moderate; diffusion limited	Low	Variable; influenced by extraction time, solvent choice, and diffusion limitations	Apoptosis induction (Caspace-3), cytotoxicity, Bax up-regulation and Bcl-2 down-regulation and antiproliferation	Extraction duration and solvent choice	Time-consuming and diffusion-limited extraction. Required purification	[2,9,11,18,19,39,40,41,47,48,54,73,74,75,76,77,78,79,80,81,82,83,84,85,86,87,88,89,90,91,92]
Soxhlet	Continuous reflux and repeated solvent cycling enabling exhaustive extraction	High (reflux)	Moderate (24–72 h)	Alkaloids, terpenoids, fatty acids, flavonoids and phenolics	Low selectivity due to exhaustive extraction and requires further separation and purification	High (Exhaustive)	High	Strong to weak but inconsistent due to exhaustive extraction and thermal effects	Apoptosis induction, cell inhibition and cell cycle arrest (G2/M)	Thermal exposure and number of extraction cycles	Thermal degradation and low selectivity due to exhaustive extraction. Further purification required	[20,22,34,35,37,38,49,89,100,101]
Decoction	Boiling-induced extraction with thermal disruption and transformation	High	Short (10–20 min)	Flavonoids, phytosterols, phenolics and polyphenols	High selectivity for water soluble compounds with enhanced recovery due to heat	Moderate; enhanced by heat for polar compounds	Moderate–high	Weak; largely limited to polar compounds and influenced by thermal transformation	Apoptosis induction, ROS generation and antiproliferation	Plant part and heat-induced chemical transformation	Limited to water-soluble compounds and potential thermal degradation	[85,90,99]
Infusion	Short-term hot water extraction of soluble compounds	Moderate–high	Short (5–30 min)	Phenolics, iridoids, flavonoids and terpenoids	High selectivity for water soluble compounds	Low–moderate; limited by short duration	Moderate	Generally weak; restricted to soluble compounds and short extraction time	Antiproliferation	Limited extraction efficiency and compound solubility	Low extraction efficiency and restricted phytochemical range	[33,52,97,98]
Hydro-distillation	Steam-driven volatilization and condensation of volatile compounds	High	Short (2–5 h)	Terpenoids	High selectivity for volatile compounds	Moderate; limited to volatile compounds	Moderate	Variable: may show strong activity for volatile-rich extracts but limited to volatile compounds	Apoptosis induction, ROS generation	Composition of volatile compounds and plant species	Limited to volatile compounds and loss of non-volatile bioactives	[16,17]

## Data Availability

No new data were created or analyzed in this study. Data sharing is not applicable to this article.
